# Transcriptional Regulation of *De Novo* Lipogenesis by SIX1 in Liver Cancer Cells

**DOI:** 10.1002/advs.202404229

**Published:** 2024-09-11

**Authors:** Ling Li, Xiujuan Zhang, Guang Xu, Rui Xue, Shuo Li, Shumeng Wu, Yuanjun Yang, Yanni Lin, Jing Lin, Guoxiao Liu, Shan Gao, Youzhi Zhang, Qinong Ye

**Affiliations:** ^1^ Beijing Institute of Biotechnology Beijing 100071 China; ^2^ School of Traditional Chinese Medicine Capital Medical University Beijing 100069 China; ^3^ Beijing Institute of Pharmacology and Toxicology Beijing 100850 China; ^4^ School of Basic Medical Sciences Shanxi Medical University Taiyuan 030000 China; ^5^ Department of Clinical Laboratory The Fourth Medical Center of PLA General Hospital Beijing 100037 China; ^6^ Department of General Surgery The First Medical Center of PLA General Hospital Beijing 100853 China; ^7^ Zhongda Hospital School of Life Sciences and Technology Advanced Institute for Life and Health Southeast University Nanjing 210096 China

**Keywords:** *de novo* lipogenesis, noncoding RNA, SIX1, transcription factor, tumor growth and metastasis

## Abstract

*De novo* lipogenesis (DNL), a hallmark of cancer, facilitates tumor growth and metastasis. Therapeutic drugs targeting DNL are being developed. However, how DNL is directly regulated in cancer remains largely unknown. Here, transcription factor sine oculis homeobox 1 (SIX1) is shown to directly increase the expression of DNL‐related genes, including ATP citrate lyase (ACLY), fatty acid synthase (FASN), and stearoyl‐CoA desaturase 1 (SCD1), via histone acetyltransferases amplified in breast cancer 1 (AIB1) and lysine acetyltransferase 7 （HBO1/KAT7）, thus promoting lipogenesis. SIX1 expression is regulated by insulin/lncRNA DGUOK‐AS1/microRNA‐145‐5p axis, which also modulates DNL‐related gene expression as well as DNL. The DGUOK‐AS1/microRNA‐145‐5p/SIX1 axis regulates liver cancer cell proliferation, invasion, and metastasis in vitro and in vivo. In patients with liver cancer, SIX1 expression is positively correlated with DGUOK‐AS1 and SCD1 expression and is negatively correlated with microRNA‐145‐5p expression. DGUOK‐AS1 is a good predictor of prognosis. Thus, the DGUOK‐AS1/microRNA‐145‐5p/SIX1 axis strongly links DNL to tumor growth and metastasis and may become an avenue for liver cancer therapeutic intervention.

## Graphical abstract

This study demonstrates that transcription factor SIX1 regulates de novo lipogenesis (DNL) in liver cancer by recruiting histone acetyltransferases AIB1 and HBO1 to DNL‐related gene promoters. SIX1 is regulated by insulin/lncRNA DGUOK‐AS1/microRNA‐145‐5p axis, which also modulates DNL‐related gene expression. DGUOK‐AS1/microRNA‐145‐5p/SIX1 axis strongly links DNL to tumor growth and metastasis and may become an avenue for liver cancer therapy.

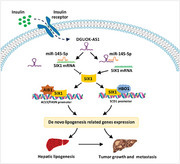

## 1 Introduction

*De novo* lipogenesis (DNL) is a complex and highly regulated metabolic pathway. DNL converts carbohydrates into fatty acids used for synthesizing triglycerides (TGs) and cholesterol, which are esterified and stored in lipid droplets (LDs).^[^ ^1^ ^]^ Dysregulation in the lipogenic pathway is associated with various metabolic anomalies, such as obesity, non‐alcoholic fatty liver disease (NAFLD), and metabolic syndrome. In addition, elevated de novo lipogenesis is considered to be a crucial factor in cancer development, and cancer therapeutic agents targeting DNL are being developed.^[^ ^2^, ^3^ ^]^ DNL includes a coordinated series of enzymatic reactions.^[^ ^1^ ^]^ The first step of this series of reactions is the conversion of citrate to acetyl‐CoA by ATP‐citrate lyase (ACLY). The resulting acetyl‐CoA is carboxylated to malonyl‐CoA by acetyl‐CoA carboxylase (ACACA/ACC1). Fatty acid synthase (FASN) is the key rate‐limiting enzyme that converts malonyl‐CoA into palmitate. Palmitate can be elongated to become stearate, and both palmitate and stearate can be converted into palmitoleate and oleate by stearoyl CoA desaturase 1 (SCD1), the enzyme controlling the conversion of saturated fatty acids (SFA) into monounsaturated fatty acids (MUFA). Palmitoleate and oleate are preferentially esterified into TGs for storage in LDs.^[^ ^4^ ^]^ Overexpression of the lipogenic enzymes, including ACLY, ACC1, FASN, and SCD1, has been widely shown in many types of cancers and is associated with poor clinical outcomes in cancer patients.

Transcription factors play a direct role in the regulation of DNL. The transcription factors sterol regulatory element‐binding protein‐1c (SREBP‐1c), carbohydrate response element‐binding protein (ChREBP), upstream stimulatory factors (USFs), liver X receptors (LXRs), and peroxisome‐proliferation‐activated receptors (PPARs) play critical roles in regulating this process.^[^ ^5^, ^6^, ^7^, ^8^, ^9^, ^10^ ^]^ SREBP1c, a master transcriptional factor regulating DNL, promotes the transcription and expression of *ACLY*, *ACC1*, *FASN*, and *SCD1*.^[^ ^5^, ^6^ ^]^ As a key transcriptional regulator, ChREBP also increases *ACLY*, *ACC1*, *FASN*, and *SCD1* expression.^[^ ^6^ ^]^ USFs are transcription factors able to bind the CANNTG sequence of FASN promoter.^[^ ^7^ ^]^ LXRs, including LXRα and LXRβ, have been reported to play an important role in regulating fatty acid synthesis. LXRs can activate lipogenic enzymes directly or through SREBP‐1c.^[^ ^8^, ^9^, ^10^ ^]^ Although a few transcription factors have been shown to directly control DNL, transcriptional regulation of DNL in cancer remains largely unknown.

Transcription factor sine oculis homeobox 1 (SIX1) is a key regulator of organogenesis and tumorigenesis.^[^ ^11^, ^12^, ^13^, ^14^, ^15^, ^16^, ^17^ ^]^ SIX1 is overexpressed in many cancers, such as hepatocellular carcinoma, breast cancer, colorectal cancer, and prostate cancer.^[^ ^14^, ^15^, ^16^ ^]^ Increased expression of SIX1 predicts poor clinical outcomes. SIX1 promotes tumor growth and metastasis through regulation of cell‐cycle progression and epithelial‐mesenchymal transition.^[^ ^18^, ^19^, ^20^ ^]^ Recently, SIX1 has attracted great attention in its ability to modulate energy metabolism.^[^ ^21^, ^22^ ^]^ SIX1 can directly promote the transcription of glycolytic genes, thereby promoting glycolysis.^[^ ^21^ ^]^ Very recently, SIX1 has been reported to activate the expression of *LXRα* and *LXRβ*, thus inducing DNL and NAFLD progression.^[^ ^22^ ^]^ However, it remains unclear whether SIX1 regulates DNL through directly regulating lipogenic gene expression and how SIX1 is regulated in the process of DNL. In this study, we show that SIX1 directly increases lipogenic gene expression through the histone acetyltransferases HBO1 and AIB1, independently of LXRα and LXRβ. In addition, we found that the insulin/lncRNA DGUOK‐AS1/microRNA‐145‐5p (miR‐145‐5p) axis controls SIX1 expression, DNL, and tumor growth and metastasis.

## 2 Results

### 2.1 Identification and Characterization of SIX1 as a Key Regulator of DNL‐Related Gene Expression

**Figure 1:**
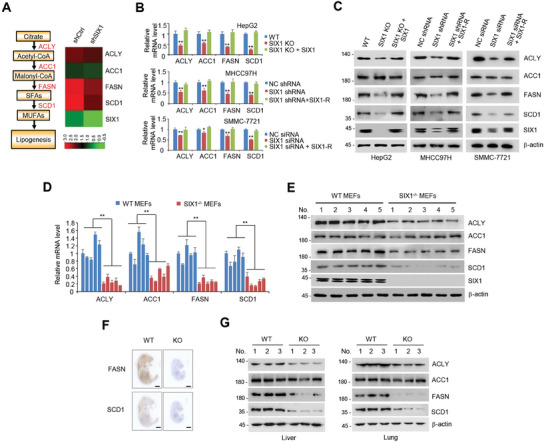
SIX1 regulates the expression of lipogenic genes. A) Heatmap of SIX1 and *de novo* lipogenic genes identified by RNA‐seq using ZR75‐1 cells stably infected with lentivirus carrying SIX1 shRNA or control shRNA. A schematic diagram of *de novo* lipogenesis pathway is shown on the left. SFAs, saturated fatty acids. MUFAs, monounsaturated fatty acids. B, C) qRT‐PCR (B) and immunoblot (C) analysis of *de novo* lipogenic gene expression in SIX1 WT or KO HepG2 cells, MHCC97H cells stably transfected with negative control shRNA (NC shRNA) or SIX1 shRNA or SMMC‐7721 cells transfected with negative control siRNA (NC siRNA) or SIX1 siRNA. The KO or KD effects were rescued by reexpression of SIX1 or shRNA‐resistant SIX1 (SIX1‐R) as indicated. β‐actin was used as a loading control. Data shown are mean ± SD of triplicate measurements. Experiments have been repeated 3 times with similar results. ^**^ *p* < 0.01 versus corresponding WT or NC shRNA cells. D, E) qRT‐PCR (D) and immunoblot (E) of lipogenic gene expression in *SIX1* WT or KO MEFs isolated from corresponding mice. The WT mice were littermates of the KO mice (*n* = 5). Data shown are mean ± SD of triplicate measurements. Experiments have been repeated 3 times with similar results. A two‐sided Student's t‐test was used to compare the means of the 2 groups. When more than 2 groups were compared, one‐way ANOVA was performed. ^*^ *p* < 0.05, ^**^ *p* < 0.01 versus corresponding control. F) Representative whole‐mount immunohistochemical staining of FASN and SCD1 for *SIX1* WT and KO mouse embryos at day 15.5 of gestation. Scale bar, 2 mm. G) Representative immunoblot analysis of lipogenic gene expression in livers or lungs from *SIX1* WT and KO mouse embryos at day 15.5 of gestation.

To identify the potential role of SIX1 in DNL, we sought data from RNA‐sequencing (RNA‐seq) (GSE93925) and found that SIX1 KD (knockdown) altered the expression of 4 DNL‐related genes (ACLY, ACC1, FASN, and SCD1) (**Figure** **1**A). To determine whether 6 family members, including SIX1‐6, are involved in DNL, we tested the effects of SIX1‐6 on DNL‐related gene expression by qRT‐PCR (quantitative reverse transcription‐polymerase chain reaction) and immunoblot. Overexpression of SIX1‐6 increased the expression of one to three DNL‐related genes at the transcriptional and protein level, with SIX1 showing the strongest ability to increase lipogenic gene expression (Figure S1A,B, Supporting Information). Except for ACC1, SIX1 overexpression increased protein expression of ACLY, FASN, and SCD1 similar to overexpression of SREBP1 (Figure S1C, Supporting Information), a well‐known master regulator of DNL.^[^ ^5^, ^6^ ^]^ Thus, we chose SIX1 for further study. SIX1 promotion of lipogenic gene expression was not dependent on SREBP1, because SREBP1 KD had no effect on enhancement of lipogenic gene expression by SIX1 overexpression (Figure S1D,E, Supporting Information). A recent study shows that SIX1 directly activated the expression of LXRs, thus inducing DNL.^[^ ^22^ ^]^ Therefore, we tested whether SIX1 promotion of lipogenic gene expression depends on LXRs. Inhibition of LXRs activities by SR‐9243 or KD of LXRα and LXRβ had no effect on the enhancement of lipogenic gene expression by SIX1 overexpression (Figure S1F–I, Supporting Information), suggesting that SIX1 promotion of the lipogenic gene expression is not dependent on LXRs.

Contrary to SIX1 overexpression, *SIX1* KO (knockout) in HepG2 liver cancer cells and SIX1 KD in MHCC97H and SMMC‐7721 liver cancer cells reduced mRNA expression of *ACLY*, *ACC1*, *FASN*, and *SCD1*, and protein expression of ACLY, FASN, and SCD1 (Figure 1B). The effect of *SIX1* KO or SIX1 KD on DNL‐related gene expression could be rescued by SIX1 reexpression (Figure 1C). Similar results were observed in *SIX1* KO mouse embryonic fibroblasts (MEFs), *SIX1* KO embryos, and liver and lung tissues from *SIX1* KO embryos (Figure 1D–G). Taken together, these data indicate that SIX1 is a key regulator of DNL‐related gene expression.

### 2.2 SIX1 Binds SIX1‐Responsive Elements to Promote Lipogenic Gene Promoter Activity

**Figure 2:**
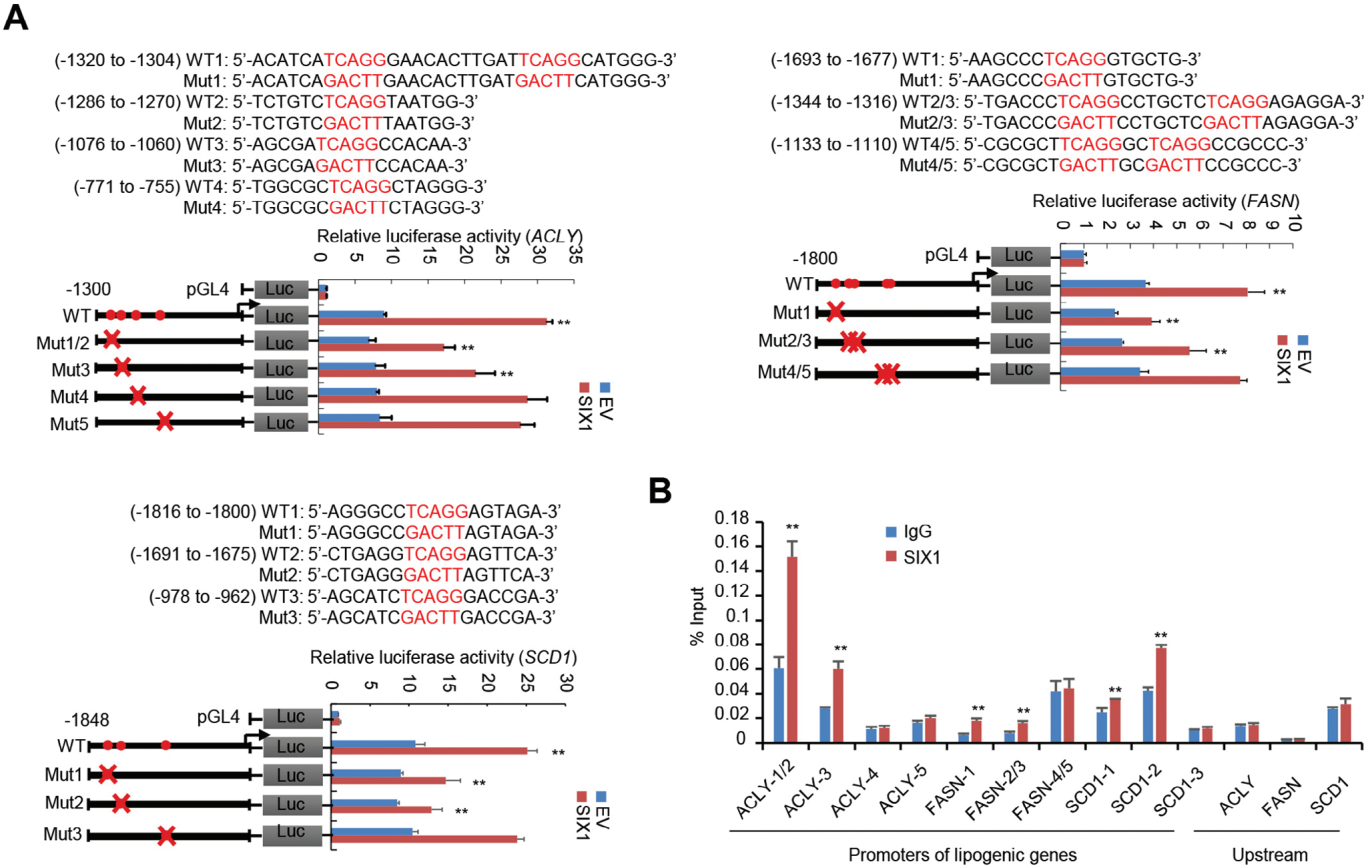
SIX1 binds the SIX1‐responsive element to enhance lipogenic gene promoter activity. A) Luciferase activity of different lipogenic gene promoter reporters in HepG2 cells transfected with FLAG‐tagged SIX1 or EV (empty vector). Filled circles show the position of the putative SIX1‐binding sites, and the “X” shows the mutated SIX1‐binding sites. The red letters of each binding region indicate the putative SIX1‐binding sequences or the mutated SIX1‐binding sequences. WT, wild‐type. Mut, mutant. B) ChIP analysis of SIX1 occupancy on promoters of lipogenic genes in HepG2 cells. IgG, normal serum. The different numbers after each gene represent the regions containing different putative SIX1‐binding sites from left to right shown in (A). Data shown are mean ± SD of triplicate measurements. Experiments have been repeated 3 times with similar results. Data were analyzed using a two‐tailed Student's t‐test. ^**^ *p* < 0.01 versus respective promoter‐reporter with EV (A). ^**^ *p* < 0.01 versus respective normal IgG (B).

To test if SIX1 transcriptionally regulates DNL‐related gene expression, we searched up to ≈2 kb of promoter regions of these genes for putative SIX1 binding sites (TCAGG)^[^ ^23^, ^24^ ^]^ and constructed promoter reporters containing the putative binding sites. SIX1 overexpression increased the reporter activity of ACLY, FASN, and SCD1 promoters. For the ACLY promoter‐reporter, mutation of putative binding sites 1 and 2 or 3, but not 4 or 5, reduced SIX1‐mediated reporter activities (**Figure** **2**A; Figure S2A, Supporting Information). For the FASN promoter‐reporter, mutation of putative binding sites 2 and 3 or 1, but not 4 and 5, attenuated activities mediated by SIX1. For the SCD1 promoter‐reporter, mutation of putative binding site 1 or 2 decreased SIX1‐mediated reporter activity. ChIP (Chromatin immunoprecipitation) assay indicated that endogenous SIX1 was recruited to the regions containing the binding sites whose mutation reduced SIX1‐mediated enhancement of the promoter‐reporter activity, but not the binding sites whose mutation did not alter that of the promoter‐reporter activity or the regions upstream of the promoters (Figure 2B; Figure S2B, Supporting Information). These data suggest that SIX1 promotes DNL‐related gene transcription by binding to these gene promoters.

### 2.3 SIX1 Promotes DNL‐Related Gene Expression through Histone Acetyltransferases AIB1 and HBO1

**Figure 3:**
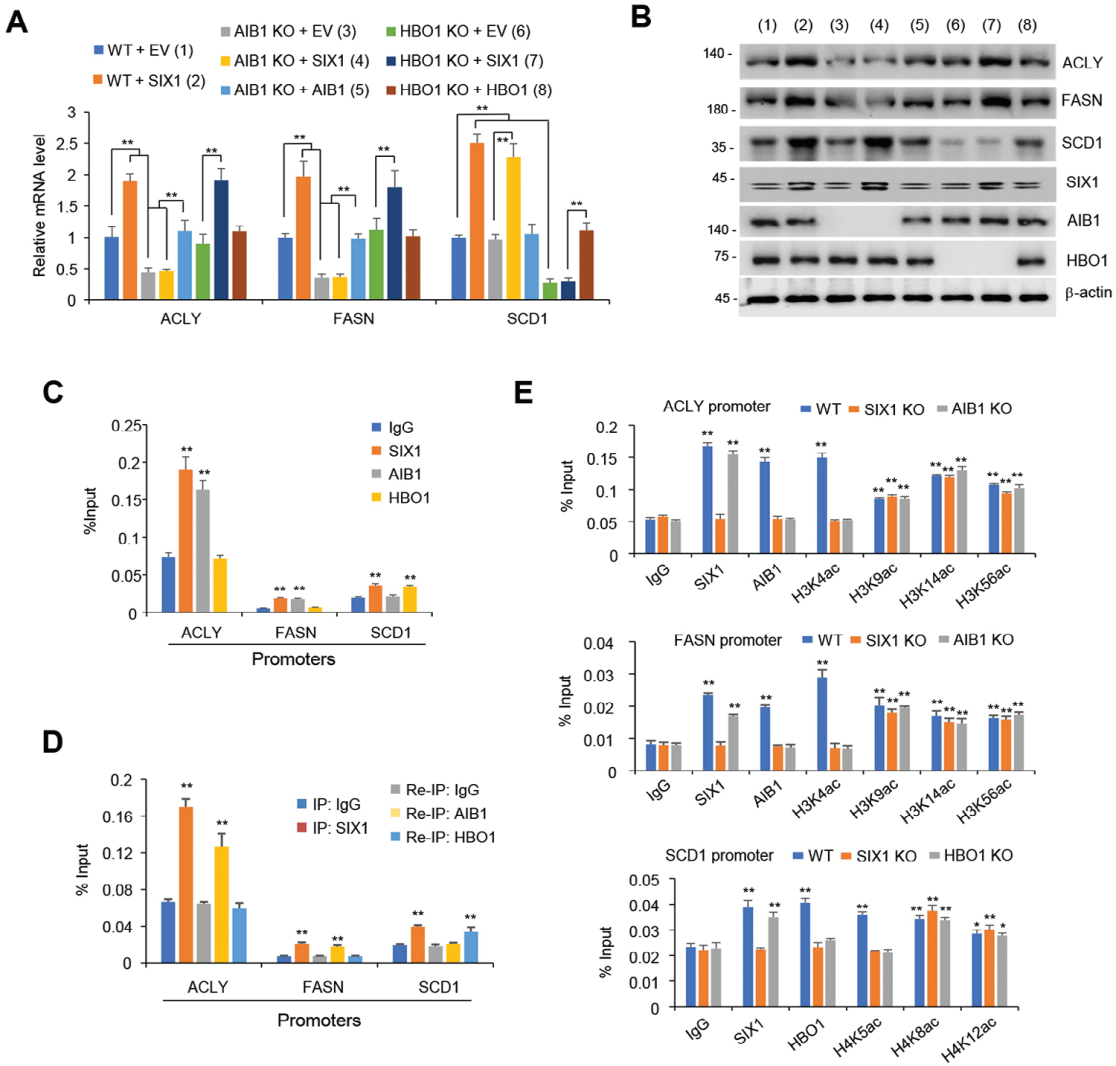
SIX1 promotes lipogenic gene expression through association with AIB1 and HBO1. A, B) qRT‐PCR (A) and immunoblot (B) analysis of *AIB1/HBO1* WT or KO HepG2 cells transfected with SIX1, HBO1, AIB1, or EV as indicated. C) ChIP analysis of SIX1, AIB1, and HBO1 occupancy on the promoters of lipogenic genes in HepG2 cells. Promoter regions of each gene represent the region containing the first or second SIX1 binding site shown in Figure 2B within the gene promoters analyzed. D) Re‐ChIP analysis of the occupancy of SIX1 and AIB1 or HBO1 on the indicated lipogenic gene promoters in HepG2 cells. E) ChIP analysis of SIX1, HBO1, AIB1, and histone H3 or H4 acetylation (ac) occupancy on the indicated promoters of lipogenic genes in *SIX1*, *AIB1*, or *HBO1* KO HepG2 cells. Data shown are mean ± SD of triplicate measurements. Experiments have been repeated 3 times with similar results. A two‐sided Student's t‐test was used to compare the means of 2 groups. When more than 2 groups were compared, one‐way ANOVA was performed. ^**^ *p* < 0.01 versus respective WT HepG2 cells transfected with empty vector (A). ^*^ *p* < 0.05, ^**^ *p* < 0.01 versus respective normal IgG (C‐E).

Histone‐modifying enzymes are required for transcription factors to regulate gene transcription, and histone acetylation, which plays a key role in transcriptional activation, is regulated by histone acetyltransferases.^[^ ^25^, ^26^ ^]^ Since our previous study shows that SIX1 interacts with AIB1/HBO1 acetyltransferases to promote glycolytic gene transcription,^[^ ^21^ ^]^ we tested whether SIX1 regulates DNL related gene transcription via AIB1 and HBO1. In HepG2 or MHCC97H cells, *AIB1* KO or AIB1 KD decreased mRNA and protein expression of ACLY and FASN, but not SCD1 and *HBO1* KO or HBO1 KD decreased that of SCD1, but not ACLY and FASN (**Figure** **3**A,B; Figure S3A,B, Supporting Information). Importantly, *AIB1/HBO1* KO or AIB1/HBO1 KD abolished the ability of SIX1 to promote the expression of the corresponding lipogenic genes. The effect of *AIB1/HBO1* KO or AIB1/HBO1 KD on lipogenic gene expression could be rescued by AIB1 or HBO1 reexpression in *AIB1/HBO1* KO HepG2 or AIB1/HBO1 KD MHCC97H cells, respectively (Figure 3A,B; Figure S3A,B, Supporting Information). These data suggest that SIX1 promotes DNL‐related gene expression through AIB1 and HBO1.

Next, we tested how SIX1 regulates lipogenic gene transcription through AIB1 and HBO1. Like SIX1, AIB1, which acetylates histones H3 and H4, especially H3, was recruited to *ACLY* and *FASN* promoters, and HBO1, which acetylates histone H4 lysine 5 (H4K5), H4K8, and H4K12,^[^ ^27^ ^]^ was recruited to the *SCD1* promoter (Figure 3C; Figure S3C, Supporting Information). Re‐ChIP experiments showed that SIX1 was associated with AIB1 or HBO1 on the corresponding promoters (Figure 3D; Figure S3D, Supporting Information). *SIX1* KO or SIX1 KD abrogated or decreased recruitment of AIB1 and acetylation of H3K4ac, but not H3K9ac, H3K14ac, and H3K56ac, to *ACLY* and *FASN* promoters, and *SIX1* KO or SIX1 KD abolished or reduced recruitment of HBO1 and acetylation of H4K5 (H4K5ac), but not H4K8ac and H4K12ac, to the *SCD1* promoter (Figure 3E; Figure S3E, Supporting Information). *AIB1* KO or AIB1 KD led to a dramatic decrease in recruitment of H3K4ac, but not H3K9ac, H3K14ac, and H3K56ac, to the SIX1/AIB1 binding sites of *ACLY* and *FASN* promoters, and *HBO1* KO or HBO1 KD caused a marked reduction of recruitment of H4K5ac, but not H4K8ac and H4K12ac, to the SIX1/HBO1 binding sites of the *SCD1* promoter. *AIB1* or *HBO1* KO had no effect on the recruitment of SIX1 to these binding sites (Figure 3E; Figure S3E, Supporting Information). Consistent with the results using liver cancer cell lines, SIX1 bound to the DNL‐related gene promoters and altered the promoter epigenotypes by recruiting AIB1 and HBO1 in liver cancer tissues (Figure S3F, Supporting Information). Taken together, these data suggest that SIX1 promotes lipogenic gene transcription through AIB1‐mediated H3K4ac or HBO1‐mediated H4K5ac.

### 2.4 SIX1 is an Insulin Responsive Gene and Stimulates Lipogenesis

**Figure 4:**
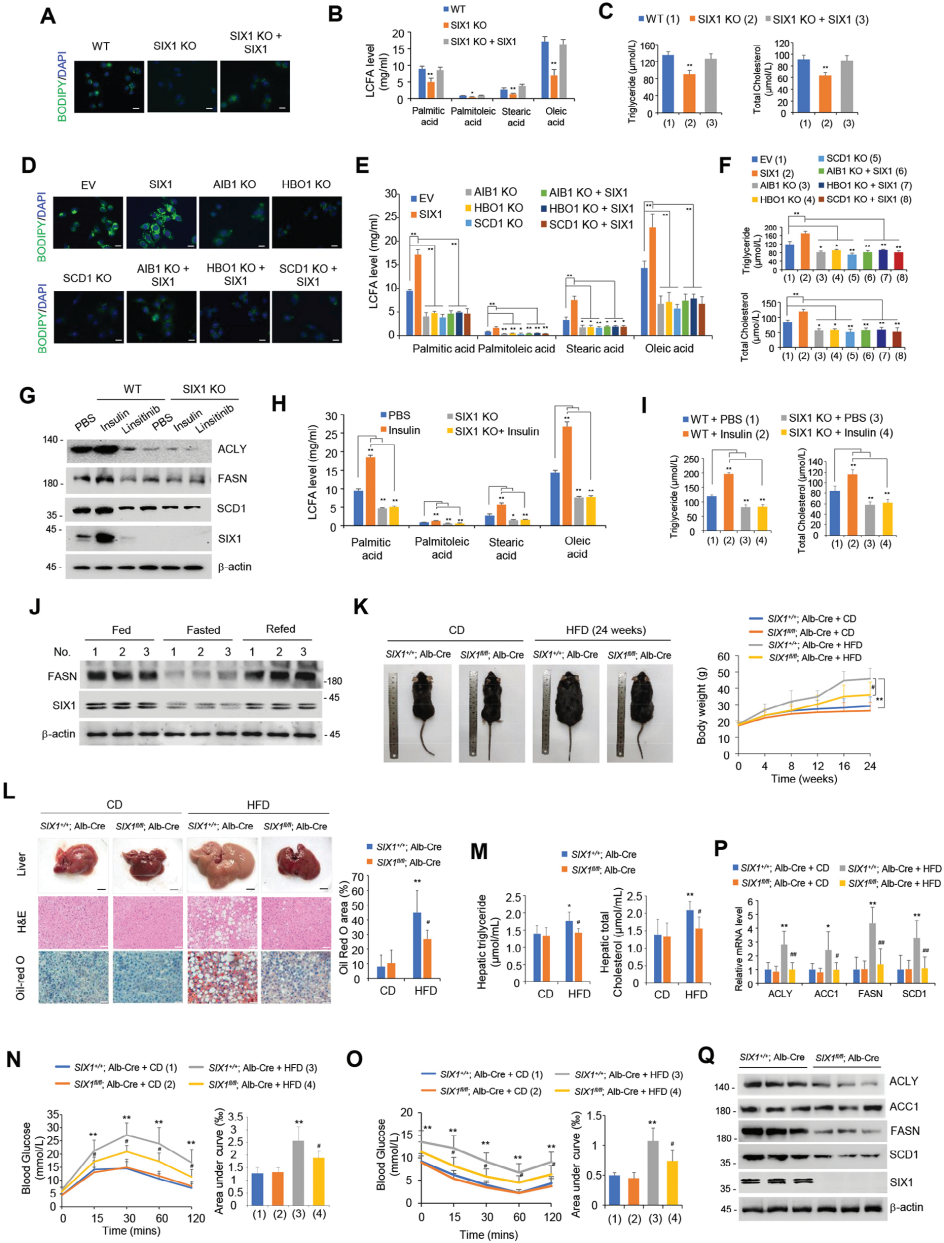
SIX1 is an insulin‐responsive gene and stimulates *de novo* lipogenesis. A) Lipid droplets (LDs) were visualized using BODIPY 493/503 in *SIX1* WT or KO HepG2 cells or *SIX1* KO HepG2 cells transfected with SIX1. Scale bar, 20 µm. B) Long‐chain fatty acid levels were analyzed by liquid chromatography‐mass spectrometry in cells from (A). C) Triglyceride and total cholesterol levels were measured in cells from (A). D‐F) Lipid droplets (D), long‐chain fatty acid levels (E), and triglyceride and total cholesterol levels (F) were analyzed in WT or *HBO1/AIB1/SCD1* KO HepG2 cells transfected with SIX1 or EV. G) Immunoblot analysis of *SIX1* WT or KO HepG2 cells treated with insulin (100 nM) or linsitinib (1.0 µM). H, I) Long‐chain fatty acid levels (H) and triglyceride and total cholesterol levels (I) were analyzed in cells from (G). J) C57BL/6J mice were fed a normal diet (Fed), subjected to fasting (Fasted) or fasted, and then refed (Refed). Livers were collected and analyzed by immunoblots. K) Representative mouse images (left) and body weight (right) of *SIX1^fl/fl^*; Alb‐Cre mice and *SIX1^+/+^*; Alb‐Cre littermates fed either chow diet (CD) or a high‐fat diet (HFD) for 24 weeks (*n* = 6). L) Representative images of liver morphology (top) and liver sections stained with H&E (middle) or Oil Red O (bottom) are shown in mice from (K). Scale bar, 5 mm for liver. Scale bar, 50 µm for H&E and Oil Red O. Quantification of the Oil Red O staining area in liver sections is shown (right). M) The hepatic triglyceride (left) and total cholesterol (right) levels in mice from (K). N) A glucose tolerance test was performed in 23‐week‐old mice from (K). Blood glucose levels were examined (left). The area under the curve was calculated (right). O) An insulin tolerance test was performed in 24‐week‐old mice from (K). Blood glucose levels were examined (left). The area under the curve was calculated (right). P, Q) Representative qRT‐PCR (P) and immunoblot (Q) analysis of the livers of the CD and HFD groups from (K). Data shown are mean ± SD of triplicate measurements (B, E, H). Data shown are mean ± SD of quintuplicate measurements (C, F, I). Experiments have been repeated 3 times with similar results. A two‐sided Student's t‐test was used to compare the means of the 2 groups. When more than 2 groups were compared, one‐way ANOVA was performed. *p < 0.05, **p < 0.01 (B, C, E, F, H, I). *p < 0.05, **p < 0.01 versus CD group (L‐O). ^#^ *p* < 0.05, ^##^ *p* < 0.01 versus SIX1^+/+^; Alb‐Cre HFD group (L‐O).

Since SIX1 promotes lipogenic gene expression, we tested whether SIX1 modulates lipogenesis in cultured cells. *SIX1* KO or SIX1 KD reduced lipid droplets (LDs) and long‐chain fatty acid (LCFA), triglyceride (TG), and total cholesterol (TC) levels in HepG2 and MHCC97H cells (**Figure** **4**A–C; Figure S4A–C, Supporting Information). These effects were reversed by SIX1 reexpression in *SIX1* KO or SIX1 KD cells. As SIX1 promotes lipogenic gene expression via AIB1 and HBO1, we tested whether SIX1 modulation of these phenotypes depends on AIB1 and HBO1. Consistent with AIB1/HBO1 regulation of lipogenic gene expression, *AIB1/HBO1* KO or AIB1/HBO1 KD caused reduced LDs and LCFA, TG, and TC levels (Figure 4D–F; Figure S4D–F, Supporting Information). As expected, *SCD1* KO or SCD1 KD decreased LDs and LCFA, TG, and TC levels. Importantly, *AIB1/HBO1/SCD1* KO or AIB1/HBO1/SCD1 KD abolished the ability of SIX1 to regulate these effects (Figure 4D–F; Figure S4D–F, Supporting Information), suggesting that SIX1 promotes LDs and LCFA, TG and TC levels through AIB1, HBO1 and SCD1.

Increased DNL and excessive TG accumulation in the liver caused by metabolic alterations are hallmarks of NAFLD pathology and are strongly associated with obesity, insulin resistance, and type 2 diabetes.^[^ ^28^, ^29^, ^30^ ^]^ To identify whether SIX1 is responsive to the insulin signal, we analyzed the expression of SIX1 in hepatocytes. Interestingly, in HepG2 and MHCC97H cells, insulin markedly increased SIX1 protein levels, accompanied by increased ACLY, FASN, and SCD1 protein levels, whereas the insulin‐like growth factor 1 receptor/ insulin receptor inhibitor linsitinib repressed these effects (Figure 4G; Figure S4G, Supporting Information). *SIX1* KO or SIX1 KD abolished the ability of insulin and linsitinib to regulate ACLY, FASN, and SCD1 expression. Moreover, *SIX1* KO or SIX1 KD abrogated insulin‐mediated enhancement of LCAF, TG, and TC levels in hepatocytes (Figure 4H,I; Figure S4H,I, Supporting Information), indicating that SIX1 is required for insulin‐mediated lipogenesis in vitro.

To explore whether SIX1 regulates lipogenesis in vivo, we first tested SIX1 and FASN expression in the liver from mice fed a normal diet, subjected to fasting or fasting, and then refed. Strikingly, like FASN expression, SIX1 expression was repressed by nutrient deprivation and induced by nutrient availability (Figure 4J). To determine the physiological function of SIX1, we moved our analyses to a high‐fat diet (HFD)‐induced NAFLD mouse model. Liver‐specific *SIX1*‐deficient (*SIX1^fl/fl^*; Alb‐cre) mice and their wild‐type (WT) (*SIX1^+/+^*; Alb‐cre) littermates were placed on a chow diet or an HFD for 24 weeks. In terms of body weight, histocellular structure, lipogenesis, and lipogenic gene expression, compared with WT mice, liver‐specific *SIX1* KO mice did not show significant changes under the normal diet condition (Figure 4K–O). However, liver‐specific *SIX1* deficiency repressed HFD‐induced obesity and body weight gain (Figure 4K), NAFLD (Figure 4L), and hepatic TG and TC levels (Figure 4M). Moreover, liver‐specific *SIX1* KO mice exhibited decreased glucose tolerance and insulin tolerance compared with their respective WT mice after HFD feeding (Figure 4N,O). Consistent with these phenotypes, liver‐specific *SIX1* KO mice demonstrated decreased lipogenic gene expression compared with WT mice after HFD feeding (Figure 4P,Q). These data support a physiological, protective role for *SIX1* deficiency in HFD‐induced obesity and NAFLD.

### 2.5 Insulin/DGUOK‐AS1/miR‐145‐5p Axis Regulates Expression of SIX1 and DNL Related Genes as well as DNL

**Figure 5:**
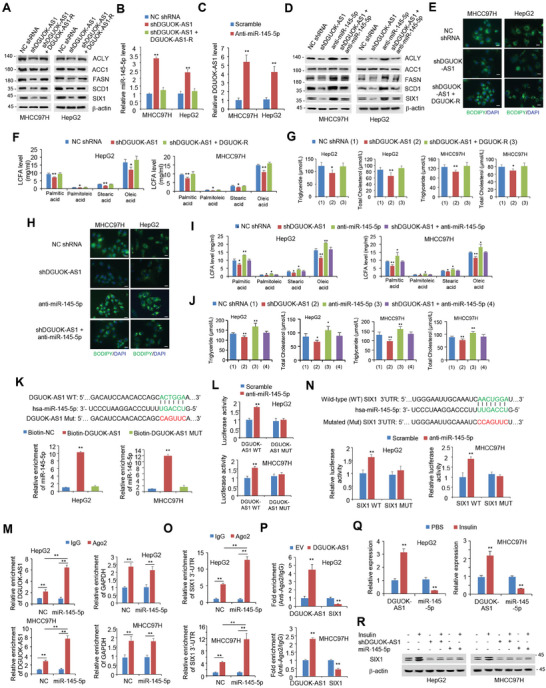
Insulin‐regulated DGUOK‐AS1/miR‐145‐5p axis modulates the expression of SIX1 and lipogenic genes as well as lipogenesis. A) Immunoblot analysis of MHCC97H and HepG2 cells stably expressing DGUOK‐AS1 shRNA (shDGUOK‐AS1) or NC shRNA or shDGUOK‐AS1‐expressing MHCC97H and HepG2 cells transfected with shRNA‐resistant DGUOK‐AS1 (DGUOK‐AS1‐R). B) qRT‐PCR analysis of miR‐145‐5p expression in cells from (A). C) qRT‐PCR analysis of DGUOK‐AS1 expression in MHCC97H or HepG2 cells transfected with scramble or anti‐miR‐145‐5p. D) Immunoblot analysis of SIX1 and *de novo* lipogenic gene expression in MHCC97H and HepG2 cells transfected with NC shRNA, shDGUOK‐AS1, anti‐miR‐145‐5p and shDGUOK‐AS1 plus anti‐miR‐145‐5p. E‐G) Analysis of lipid droplets (E), long‐chain fatty acid levels (F), and triglyceride and total cholesterol levels (G) in MHCC97H or HepG2 cells transfected as in (A). Scale bar, 20 µm (E). H‐J) Analysis of lipid droplets (H), long‐chain fatty acid levels (I), and triglyceride and total cholesterol levels (J) in MHCC97H or HepG2 cells transfected as in (D). Scale bar, 20 µm (H). K) RNA pull‐down analysis of the enrichment of miR‐145‐5p in HepG2 or MHCC97H cells using biotin‐labeled NC, DGUOK‐AS1 or its mutant (DGUOK‐AS1‐Mut). L) Luciferase activity analysis of MHCC97H and HepG2 cells transfected with WT or mutant DGUOK‐AS1 reporter and anti‐miR‐145‐5p or scramble. M) The enrichment of DGUOK‐AS1 or GAPDH in HepG2 or MHCC97H cells transfected with miR‐145‐5p or NC was determined by RIP using normal IgG or anti‐Ago2. N) miRNA luciferase reporter assays in HepG2 and MHCC97H cells transfected with WT or mutated SIX1 3′‐UTR reporter and anti‐miR‐145‐5p or scramble. The top panel indicates WT and mutant forms of putative miR‐145‐5p target sequences of SIX1 3′‐UTR. Red font indicates the mutated miR‐145‐5p‐binding sites within human SIX1 3′‐UTR. O) The enrichment of SIX1 3′‐UTR in HepG2 and MHCC97H cells transfected with miR‐145‐5p or NC was examined by RIP using normal IgG or anti‐Ago2. P) The enrichment of SIX1 3′‐UTR in HepG2 and MHCC97H cells transfected with NC, miR‐145‐5p, or miR‐145‐5p plus DGUOK‐AS1 was examined by RIP using normal IgG or anti‐Ago2. Q) qRT‐PCR analysis of the expression of DGUOK‐AS1 and miR‐145‐5p in HepG2 and MHCC97H cells treated with insulin (100 nM) for 24 h. R) Immunoblot analysis of SIX1 expression in HepG2 and MHCC97H cells transfected with shDGUOK‐AS1, miR‐145‐5p, or corresponding control and treated with insulin (100 nM) for 24 h. Values shown are mean ± SD of triplicate measurements (B, C, F, I, K‐Q). Data shown are mean ± SD of quintuplicate measurements (G, J). Experiments have been repeated 3 times with similar results. A two‐sided Student's t‐test was used to compare the means of the 2 groups. When more than 2 groups were compared, one‐way ANOVA was performed. ^*^ *p* < 0.05, ^**^ *p* < 0.01 versus respective NC or scramble.

Long noncoding RNAs (lncRNAs) play important roles in gene regulation.^[^ ^31^ ^]^ LncRNAs are also involved in a variety of cellular biological functions, such as cell differentiation, glycolysis, and lipogenesis.^[^ ^32^, ^33^, ^34^, ^35^ ^]^ To identify potential lncRNAs regulating SIX1 expression, we used TANRIC database (https://www.tanric.org/) and ENCORI database (http://starbase.sysu.edu.cn/) to screen lncRNAs which are positively correlated with SIX1 expression, are overexpressed, and predict poor prognosis in patients with hepatocellular carcinoma. We identified 3 lncRNAs, DGUOK‐AS1, LINC00460, and SLC7A11‐AS1, positively correlated with SIX1 expression, overexpressed and exhibiting poor clinical outcomes (Figure S5A–I, Supporting Information). To investigate whether the identified lncRNAs regulate SIX1 expression, we transfected liver cancer cells with each of the 3 lncRNAs. Among these lncRNAs, DGUOK‐AS1 increased SIX1 protein and mRNA expression most obviously in HepG2 cells (Figure S5J, Supporting Information). Moreover, DGUOK‐AS1 KD decreased SIX1 protein expression and expression of lipogenic genes, including *ACLY*, *FASN*, and *SCD1* (**Figure** **5**A; Figure S6A, Supporting Information). These effects were reversed by DGUOK‐AS1 reexpression in DGUOK‐AS1 KD HepG2 and MHCC97H cells. Thus, we selected DGUOK‐AS1 for further investigation.

LncRNAs located in the cytoplasm can act as miRNA sponges to regulate gene expression, and miRNAs often repress gene expression by preferentially binding to the 3′‐untranslated regions (3′‐UTRs) of target mRNAs. Subcellular localization experiments showed that DGUOK‐AS1 was predominantly localized in the cytoplasm (Figure S6B, Supporting Information). Thus, we used miRcode database to predict miRNAs that target both DGUOK‐AS1 and SIX1. Our analysis predicted several potential miRNAs, among which miR‐145‐5p repressed SIX1 protein expression most obviously in HepG2 cells (Figure S6C, Supporting Informatio). Therefore, we chose miR‐145‐5p for further study. DGUOK‐AS1 overexpression weakened miR‐145‐5p expression in HepG2 and MHCC97H cells (Figure S6D, Supporting Information). In contrast, DGUOK‐AS1 KD enhanced miR‐145‐5p expression (Figure 5B). These effects were reversed by DGUOK‐AS1 reexpression in DGUOK‐AS1 KD HepG2 and MHCC97H cells. Moreover, miR‐145‐5p inhibitor increased DGUOK‐AS1 expression (Figure 5C; Figure S6E, Supporting Information), while miR‐145‐5p mimics decreased DGUOK‐AS1 expression (Figure S6F, Supporting Information). DGUOK‐AS1 KD and miR‐145‐5p mimics decreased and miR‐145‐5p inhibitor increased expression of SIX1 and SIX1‐regulated lipogenic genes, including *ACLY*, *FASN*, and *SCD1* (Figure 5D; Figure S6G,H, Supporting Information). Importantly, miR‐145‐5p inhibition abolished the ability of DGUOK‐AS1 KD to decrease SIX1 and SIX1‐regulated lipogenic gene expression (Figure 5D). Intriguingly, in the liver tissues of mice fed a normal diet, subjected to fasting or fasting and then refed, like FASN and SIX1 mRNA expression, DGUOK‐AS1 expression was repressed and miR‐145‐5p expression was stimulated by nutrient deprivation and reversed by nutrient availability (Figure S6I, Supporting Information). Taken together, these results suggest that expression of DGUOK‐AS1 and miR‐145‐5p is reciprocally repressed and DGUOK‐AS1 promotes expression of SIX1 and SIX1‐regulated lipogenic genes via miR‐145‐5p.

Consistent with the results of DGUOK‐AS1 modulation of SIX1 and SIX1‐regulated lipogenic gene expression, DGUOK‐AS1 KD reduced LDs and LCFA, TG, and TC levels in HepG2 and MHCC97H cells (Figure 5E–G). These effects were reversed by DGUOK‐AS1 reexpression in DGUOK‐AS1 KD cells. miR‐145‐5p inhibitor increased LDs and LCFA, TG, and TC levels in HepG2 and MHCC97H cells (Figure 5H–J). Moreover, miR‐145‐5p inhibition abolished the ability of DGUOK‐AS1 KD to reduce LDs and LCFA, TG, and TC levels (Figure 5H–J). These results reveal that DGUOK‐AS1 regulates lipogenesis through miR‐145‐5p in hepatocytes.

To investigate whether DGUOK‐AS1 is a miR‐145‐5p sponge, we performed RNA pull‐down experiments. Biotin‐labeled DGUOK‐AS1, but not DGUOK‐AS1 mutant, pulled down miR‐145‐5p in HepG2 and MHCC97H cells (Figure 5K). We further confirmed the relationship between DGUOK‐AS1 and miR‐145‐5p by luciferase reporter assay in HepG2 and MHCC97H cells. miR‐145‐5p inhibitor increased the luciferase activity of WT, but not mutant, DGUOK‐AS1 reporter (Figure 5L). To investigate the potential direct binding between DGUOK‐AS1 and miR‐145‐5p, we performed RNA immunoprecipitation (RIP) assays based on Ago2, which can enrich for targets bound by miRNAs upon immunoprecipitation. In HepG2 and MHCC97H cells, DGUOK‐AS1 level precipitated by anti‐Ago2 antibody was higher than that precipitated by anti‐IgG, and miR‐145‐5p overexpression caused a significant increase in the enrichment of DGUOK‐AS1, but not glyceraldehyde‐3‐phosphate dehydrogenase (GAPDH) mRNA, an unrelated mRNA (Figure 5M). Taken together, these data suggest that DGUOK‐AS1 acts as a miR‐145‐5p sponge.

Next, we investigated the mechanism by which the DGUOK‐AS1/miR‐145‐5p axis regulates SIX1 expression. miR‐145‐5p inhibitor increased the luciferase reporter activity of SIX1 WT 3′‐UTR, but not mutated 3′‐UTR in which the binding sites for miR‐145‐5p were mutated (Figure 5N). RIP assay showed that SIX1 3′‐UTR level precipitated by anti‐Ago2 antibody in HepG2 and MHCC97H cells was higher than that precipitated by anti‐IgG, indicating that SIX1 3′‐UTR forms a complex with Ago2 (Figure 5O). Furthermore, miR‐145‐5p overexpression increased endogenous SIX1 3′‐UTR enrichment in the Ago2 complex (Figure 5O). Overexpression of DGUOK‐AS1 caused a significant decrease in the miR‐145‐5p‐mediated enrichment of SIX1 3′‐UTR pulled down by Ago2 (Figure 5P), indicating that DGUOK‐AS1 competes with miR‐145‐5p for binding to SIX1 3′‐UTR. Taken together, these results suggest that miR‐145‐5p represses SIX1 expression by directly targeting its 3′ UTR, and DGUOK‐AS1 serves as a competing endogenous RNA (ceRNA) for miR‐145‐5p to regulate SIX1 expression.

Since insulin and the DGUOK‐AS1/miR‐145‐5p axis regulate SIX1 expression, we investigated whether insulin regulates DGUOK‐AS1 and miR‐145‐5p expression. Intriguingly, insulin increased DGUOK‐AS1 expression and decreased miR‐145‐5p expression in HepG2 and MHCC97H cells (Figure 5Q). DGUOK‐AS1 KD or miR‐145‐5p overexpression attenuated the ability of insulin to stimulate SIX1 expression (Figure 5R; Figure S6J, Supporting Information), suggesting that insulin regulates SIX1 expression through DGUOK‐AS1 and miR‐145‐5p.

### 2.6 The DGUOK‐AS1/miR‐145‐5p/SIX1 Axis Regulates Liver Cancer Cell Growth and Metastasis

**Figure 6:**
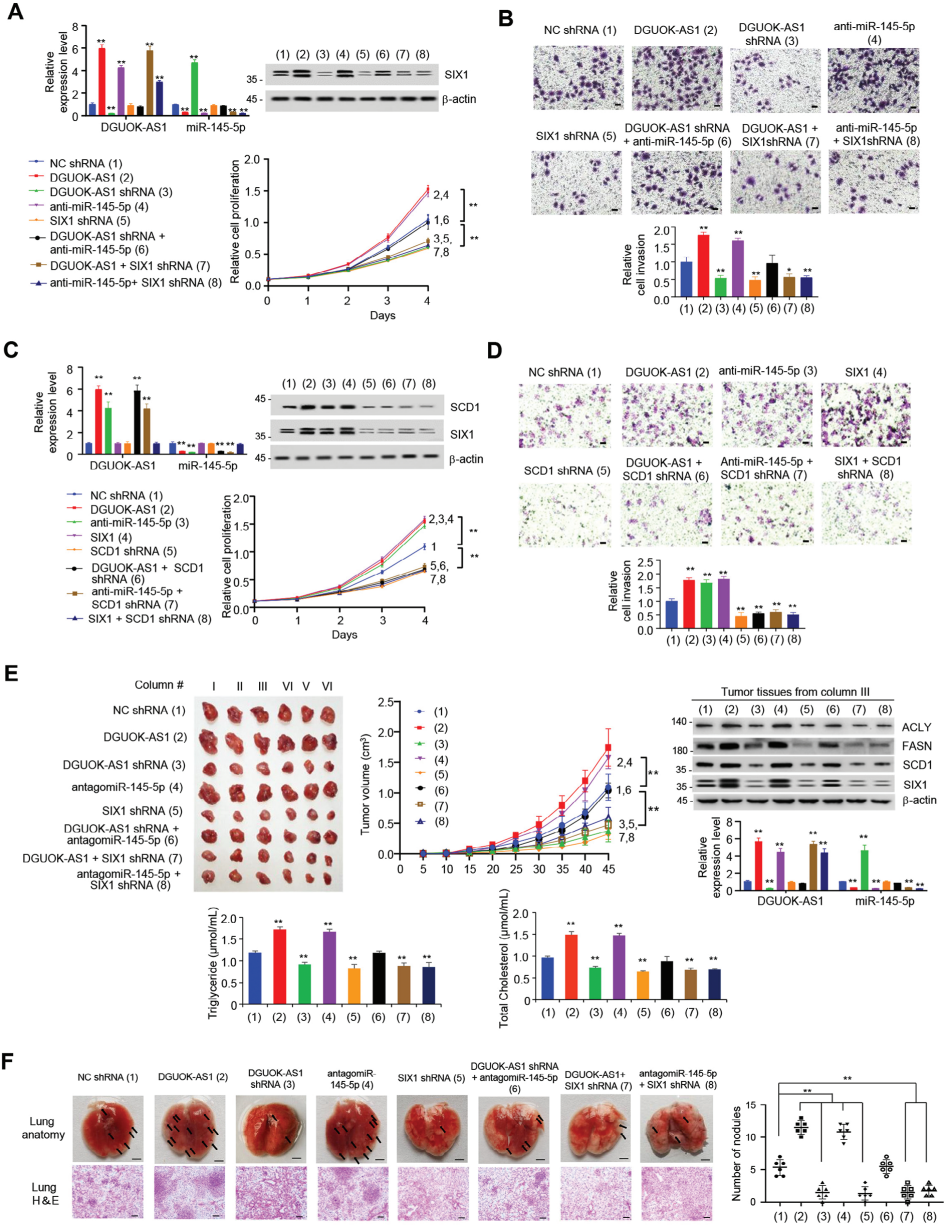
DGUOK‐AS1/miR‐145‐5p/SIX1 axis modulates HCC growth and metastasis. A) MHCC97H cells stably expressing DGUOK‐AS1 shRNA, SIX1 shRNA or NC shRNA were transfected with DGUOK‐AS1 or anti‐miR‐145‐5p, and the cell proliferation curve was then determined. B) Cell invasion assay of MHCC97H cells as described in (A). The relative cell invasions are shown in the lower panel. Scale bar, 100 µm. C) MHCC97H cells stably expressing SCD1 shRNA or NC shRNA were transfected with DGUOK‐AS1, anti‐miR‐145‐5p, or SIX1 as indicated, and the cell proliferation curve was then determined. D) Cell invasion assay of MHCC97H cells as described in (C). The relative cell invasions are shown in the lower panel. Scale bar, 100 µm. Data shown are mean ± SD of triplicate measurements (A–D). Experiments have been repeated 3 times with similar results. Statistical significance was assessed by one‐way ANOVA. ^*^ *p* < 0.05, ^**^ *p* < 0.01 versus corresponding NC shRNA. E) Tumor growth curve of MHCC97H cells stably expressing DGUOK‐AS1, DGUOK‐AS1 shRNA, SIX1 shRNA, or NC shRNA and treated with antagomiR‐145‐5p as indicated (mean ± SD; *n* = 6). ^**^ *p* < 0.01 at day 45. Images of xenograft tumors are shown in the left panel. Representative immunoblot shows expression of *de novo* lipogenesis‐related gene in representative tumor tissues. qRT‐PCR indicates the expression of DGUOK‐AS1 and miR‐145‐5p. Triglyceride and total cholesterol levels were measured in representative tumor tissues. Data shown are mean ± SD of triplicate measurements. Statistical significance was assessed by one‐way ANOVA. ^**^ *p* < 0.01 versus corresponding NC shRNA. F) Representative lung tissues and hematoxylin and eosin (H&E)‐stained sections of the lung tissues at 35 days from nude mice injected by tail vein with MHCC97‐H cells stably transfected as in (E) (*n* = 6). Scale bar, 25 mm for lung anatomy. Scale bar, 200 µm for H&E. Arrows indicate tumor foci. The number of tumor nodules spread throughout the pulmonary region is shown. Statistical testing was performed using a one‐way ANOVA. ^**^ *p* < 0.01 versus corresponding NC shRNA.

As DGUOK‐AS1/miR‐145‐5p/SIX1 axis regulates DNL, which plays an important role in modulating cancer cell proliferation and metastasis, we first examined if the DGUOK‐AS1/miR‐145‐5p/SIX1 axis regulates cancer cell proliferation and invasion in vitro. DGUOK‐AS1 overexpression or miR‐145‐5p inhibitor increased the proliferation and invasion of MHCC97H and HepG2 cells, and DGUOK‐AS1 KD or SIX1 KD/*SIX1* KO reduced their proliferation and invasion (**Figure** **6**A,B; Figure S7A,B, Supporting Information). miR‐145‐5p inhibitor abrogated the ability of DGUOK‐AS1 KD to inhibit proliferation and invasion, and SIX1 KD or *SIX1* KO abolished the ability of DGUOK‐AS1 or miR‐145‐5p inhibitor to promote proliferation and invasion (Figure 6A,B; Figure S7A,B, Supporting Information). These data suggest that the DGUOK‐AS1/miR‐145‐5p axis regulates cancer cell proliferation and invasion via SIX1.

Next, we tested whether the DGUOK‐AS1/miR‐145‐5p/SIX1 axis modulates cancer cell proliferation and invasion through lipogenic enzymes. DGUOK‐AS1 overexpression, miR‐145‐5p inhibitor or SIX1 overexpression increased the proliferation and invasion of MHCC97H and HepG2 cells, and SCD1 KD/ *SCD1* KO inhibited their proliferation and invasion (Figure 6C,D; Figure S7C,D, Supporting Information). Importantly, SCD1 KD/ *SCD1* KO almost abolished the ability of DGUOK‐AS1 overexpression, miR‐145‐5p inhibitor, or SIX1 overexpression to increase liver cancer cell proliferation and invasion, suggesting that SCD1 is critical for the regulation of cancer cell proliferation and invasion by DGUOK‐AS1/miR‐145‐5p/SIX1 axis.

Consistent with cell proliferation and invasion results in vitro, DGUOK‐AS1 overexpression and miR‐145‐5p inhibitor promoted liver tumor growth, metastasis, and TC and TG levels in nude mice, whereas DGUOK‐AS1 KD and SIX1 KD or *SIX1* KO inhibited liver tumor growth, metastasis, and TC and TG levels (Figure 6E,F; Figure S7E, Supporting Information). miR‐145‐5p inhibitor abolished the ability of DGUOK‐AS1 KD to inhibit liver tumor growth, metastasis, and TC and TG levels, and SIX1 KD or *SIX1* KO abrogated the ability of DGUOK‐AS1 and miR‐145‐5p inhibitor to promote liver tumor growth, metastasis, and TC and TG levels, suggesting that DGUOK‐AS1/miR‐145‐5p axis regulates tumor growth, metastasis and DNL via SIX1.

### 2.7 The insulin/DGUOK‐AS1/miR‐145‐5p Axis Alters the Binding of AIB1 and HBO1 to the DNL‐Related Gene Promoters in Cultured Cells and Tumor Tissues

Since the insulin/DGUOK‐AS1/miR‐145‐5p axis regulates SIX1 expression as well as liver cancer cell growth and metastasis, we investigated whether this axis affects histone acetylation at the SIX1 promoter and the recruitment of AIB1 and HBO1 to the DNL‐related gene promoters in cultured cells and liver tumor tissues. In liver cancer cells, the insulin/DGUOK‐AS1/miR‐145‐5p axis did not alter the histone acetylation at the SIX1 promoter (Figure S8A, Supporting Information), suggesting that the insulin/DGUOK‐AS1/miR‐145‐5p axis may not regulate SIX1 expression at the transcriptional level. Interestingly, the ChIP assay showed that the insulin/DGUOK‐AS1/miR‐145‐5p axis altered the binding of AIB1 and HBO1 to the DNL‐related gene promoters in cultured cells and liver tumor tissues (Figure S8B,C, Supporting Information).

### 2.8 Clinical Relevance of the DGUOK‐AS1/miR‐145‐5p/SIX1 Axis in Liver Cancer

**Figure 7:**
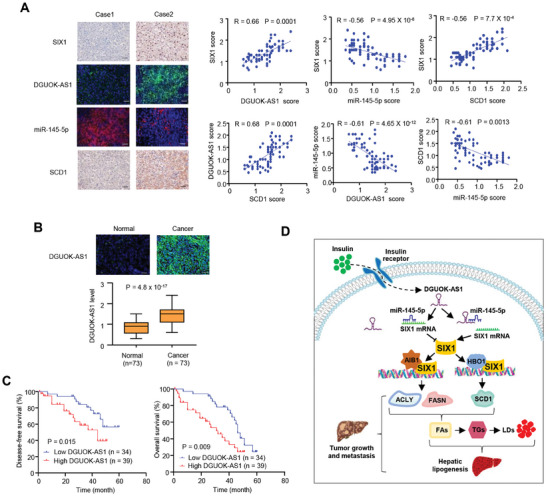
Clinical relevance of the DGUOK‐AS1/miR‐145‐5p/SIX1 axis in liver cancer. A) Representative IHC of 73 liver cancer patients. SIX1 and SCD1 were assessed by IHC, and DGUOK‐AS1 and miR‐145‐5p by FISH. Scale bar: 100 µm. The correlations among DGUOK‐AS1, miR‐145‐5p, SCD1, and SIX1 were analyzed as indicated. Case 1 and case 2 refer to 2 representative samples categorized by low and high expression of SIX1. Data was analyzed by Spearman's Rank Correlation test. B) DGUOK‐AS1 expression in 73 cancerous liver tissues and matched adjacent normal liver tissues was determined by FISH. The DGUOK‐AS1 expression levels were plotted and compared between normal and cancer tissues (Mann‐Whitney U test). C) The disease‐free and overall survival curves related to low and high expression of DGUOK‐AS1 were analyzed in 73 liver cancer patients from (A) using the Kaplan‐Meier method. D) A proposed model underlying the role of the insulin/DGUOK‐AS1/miR‐145‐5p/SIX1 axis in *de novo* lipogenesis and liver tumor growth and metastasis. Insulin stimulates the expression of DGUOK‐AS1, thus sponging miR‐145‐5p. Inhibition of miR‐145‐5p promotes expression of SIX1, which recruits histone acetyltransferases AIB1 and HBO1 to induce expression of lipogenic genes (*ACLY*, *FASN*, and *SCD1*). Induction of lipogenic gene expression promotes hepatic lipogenesis and liver tumor growth and metastasis. FAs, fatty acids. TGs, triglycerides. LDs, lipid droplets.

In liver cancer patients we analyzed, SIX1 expression was positively correlated with DGUOK‐AS1 and SCD1 expression and negatively correlated with expression of miR‐145‐5p; DGUOK‐AS1 expression was positively correlated with SCD1 expression and negatively correlated with expression of miR‐145‐5p; and SCD1 expression was negatively correlated with expression of miR‐145‐5p (**Figure** **7**A).

SIX1 is overexpressed in various human cancers, including liver cancer, and its overexpression correlates with poor clinical outcomes.^[^ ^12^, ^13^, ^14^, ^15^ ^]^ miR‐145‐5p is downregulated in many cancers, such as hepatocellular carcinoma, prostate cancer, breast cancer, and ovarian cancer,^[^ ^36^, ^37^, ^38^, ^39^ ^]^ and low miR‐145‐5p expression is associated with poor prognosis in patients with liver cancer.^[^ ^36^ ^]^ However, the clinical significance of DGUOK‐AS1 expression in liver cancer is unknown. Our analysis showed that DGUOK‐AS1 was significantly upregulated in liver cancer tissues (Figure 7B). Liver cancer patients with decreased DGUOK‐AS1 expression had longer disease‐free survival (DFS) and overall survival (OS) (Figure 7C).

## 3 Discussion

DNL plays an important role in cancer progression.^[^ ^1^ ^]^ Our study establishes the DGUOK‐AS1/miR‐145‐5p/SIX1 axis as a new regulatory pathway in DNL. The transcription factor SIX1 directly promotes the expression of key lipogenic genes that facilitate DNL as well as tumor growth and metastasis. The expression and function of SIX1 are controlled by the DGUOK‐AS1/miR‐145‐5p axis. DNL has been shown to be mainly activated by the transcription factors SREBP‐1c, ChREBP, USFs, LXRs, and PPARγ.^[^ ^3^, ^4^, ^5^, ^6^, ^7^, ^8^ ^]^ Using *SIX1* KO hepatocarcinoma cell lines, *SIX1* KO MEFs, *SIX1* KO mice embryos, and tumor samples, we show that SIX1 regulates lipogenic gene expression, LDs, LCFA synthesis, and the production of TG and TC, facilitating tumor growth and metastasis. Our data indicate that SIX1 is another key transcription factor for the regulation of DNL and also indicate a causal role for SIX1 in DNL regulation (Figure 7D).

Many studies have shown that SIX1 is overexpressed in various human cancers and its elevated expression is associated with poor clinical outcomes.^[^ ^14^, ^15^, ^16^ ^]^ SIX1 has been demonstrated to play a key role in cell metabolism, including glycolysis and hepatic lipogenesis.^[^ ^21^, ^22^ ^]^ However, upstream regulators of SIX1 in DNL are not clear. Recently, ncRNAs, such as lncRNAs and miRNAs, have been shown to regulate gene expression, cell metabolism as well as tumorigenesis and cancer progression. In this study, we found that DGUOK‐AS1 contributes to liver cancer cell growth and metastasis through miR‐145‐5p mediated modulation of SIX1 expression and SIX1‐regulated lipogenesis. In contrast to SIX1 and DGUOK‐AS1, miR‐145‐5p represses DNL by inhibiting SIX1 expression, resulting in the suppression of liver tumor growth and metastasis. Thus, we identify a DGUOK‐AS1/miR‐145‐5p axis and previously unknown roles for DGUOK‐AS1 and miR‐145‐5p in regulating DNL and the DGUOK‐AS1/miR‐145‐5p axis in modulating liver tumor growth and metastasis. Furthermore, DGUOK‐AS1 and SIX1 is stimulated by insulin, a secreted protein related to lipogenesis and cancer, but miR‐145‐5p is inhibited by insulin. Insulin regulates SIX1 expression through the DGUOK‐AS1/miR‐145‐5p axis. Mechanisms underlying insulin‐mediated induction of DGUOK‐AS1 remain to be investigated. Since the DGUOK‐AS1/miR‐145‐5p/SIX1 axis is dysregulated in cancer, correlates with prognosis, and controls DNL, and *SIX1* KO attenuates NAFLD progression, this axis is expected to be a promising therapeutic target for curing liver cancer and NAFLD.

Potentiation of DNL‐related gene transcription by SIX1 is mediated mainly through histone acetyltransferases AIB1 and HBO1. Like DGUOK‐AS1, miR‐145‐5p, and SIX1, AIB1 and HBO1 are novel regulators of lipogenic gene expression in cancer cells. SRC‐2 and SRC‐3 (also named AIB1) have been shown to concomitantly promote human adipocyte differentiation by attenuating phospho‐PPARγ S114 and modulating PPARγ cellular heterogeneity.^[^ ^40^ ^]^ Ablation of AIB1 in mice impairs adipogenesis and enhances energy expenditure.^[^ ^41^ ^]^ Many reports demonstrate strong HBO1 expression in various human cancers, such as breast cancer and gastric cancer.^[^ ^42^, ^43^, ^44^ ^]^ AIB1 is overexpressed in many cancers and its overexpression correlates with poor survival of patients.^[^ ^45^, ^46^, ^47^ ^]^ AIB1 and HBO1 have distinct roles in the regulation of lipogenic gene expression. AIB1 is required for SIX1 modulation of ACLY and FASN expression, and HBO1 is necessary for SIX1 modulation of SCD1 expression. Since KO of AIB1 or HBO1 attenuates the ability of SIX1 to promote DNL in cancer cells, SIX1 is necessary for the recruitment of AIB1 and HBO1 to lipogenic gene promoters, and DNL is important for tumor growth and metastasis, targeting SIX1 may make liver cancer therapy more effective than targeting AIB1 or HBO1.

As a transcription factor, SIX1 regulates both glycolysis and lipogenesis. There are also other transcription factors that are reported to regulate glycolysis and lipogenesis. The transcription factor ChREBP (carbohydrate‐responsive element‐binding protein), in response to increased glucose concentration, is translocated to the nucleus and activates several genes involved in glucose and lipid metabolism as well as genes involved in insulin signaling.^[^ ^48^ ^]^ Forkhead box O1 (FoxO1) transcription factor stimulates gluconeogenic gene expression and suppresses the expression of genes involved in glycolysis and lipogenesis, including glucokinase and SREBP‐1c.^[^ ^49^ ^]^

## 4 Experimental Section

### Plasmids, siRNAs, shRNAs, and Lentiviral Vectors

The eukaryotic expression vectors encoding FLAG‐ or MYC‐tagged proteins or untagged proteins were generated by inserting PCR‐amplified fragments into pcDNA3 (Invitrogen). The lipogenic gene promoter luciferase reporters were made by inserting PCR‐amplified promoter fragments from genomic DNA into the pGL4‐Basic vector (Promega). The mutants for the luciferase reporters were made by recombinant PCR. The cDNA target sequences of siRNAs and/or shRNAs for SIX1, HBO1, AIB1, LXRα, LXRβ, SCD1, DGUOK‐AS1, and SREBP1 are listed in Table S1 (Supporting Information). Lentiviral vectors for gene overexpression were obtained by inserting PCR‐amplified gene fragments into pCDH (System Biosciences). Lentiviral shRNA vectors were constructed by cloning short hairpin RNA fragments into pSIH‐H1‐Puro (System Biosciences).

### Cell Lines, Transfection and Infection

Human liver cancer cell line HepG2 and human embryonic kidney HEK293T cells were purchased from the American Type Culture Collection (Rockefeller, MD). Human liver cancer cell line MHCC97‐H (C6585) was purchased from Beyotime. Cells have previously been tested for mycoplasma contamination. Cells were cultured in Dulbecco's Modified Eagle Medium (DMEM) supplemented with 10% fetal bovine serum (FBS; Hyclone). All cell lines were maintained at 37 °C in 5% CO_2_. Plasmid and siRNAs were transfected with Lipofectamine 3000 reagent (Invitrogen, Carlsbad, CA) and RNAiMAX (Invitrogen, Carlsbad, CA), respectively. When transfected with the plasmid, cells were collected 24 h later for further analysis. Lentiviruses were produced by cotransfection of HEK293T cells with recombinant lentivirus vectors and pPACK Packaging Plasmid Mix (System Biosciences) using Megatran reagent (Origene) and were used to infect target cells according to the manufacturer's instructions. Viral supernatants were harvested 48 h after transfection, and titers were detected. The target cells were then infected with lentiviral constructs containing 8 µg mL^−1^ polybrene (Sigma‐Aldrich, Saint Louis, MO). To establish stable cell lines, infected HepG2 and MHCC97H cells were selected in 1 µg mL^−1^ puromycin and transfected HepG2 and MHCC97H cells were selected in 600 µg mL^−1^ G418.

### SIX1, HBO1, AIB1, and SCD1 Knockout (KO) Cancer Cell Lines

*SIX1*, *HBO1*, *AIB1*, and *SCD1* KO cancer cell lines were generated using the CRISPR/Cas9 system. The CRISPRs were designed using the CRISPR design web tool (www.guidescan.com). The single guide RNA (sgRNA) sequences targeted by SIX1, HBO1, AIB1, and SCD1 was CCTGCACAAGAACGAGAGCGTAC, CCGACGATCTGCTCGAGTCACCC, TGATGTATATTCAAGATGAGTGG and GCCTTCCTTATCCTTGTAGGTGG, respectively. The sgRNA was cloned into the lentiCRISPRv2 vector (Addgene #52961). Recombinant lentiviruses were produced by cotransfecting HEK293T cells with a lentiviral vector using the Megatron reagent (Origene, Rockville, MD). HepG2 and MHCC97H cells were infected with purified lentiviruses combined with 8 µg mL^−1^ polybrene (Sigma‐Aldrich, Saint Louis, MO) and then selected with 1 µg mL^−1^ puromycin. CRISPR cell lines were clonal. Rescue experiments were performed to avoid off‐target effects.

### SIX1 Conditional KO Mice

All animal experiments were performed in compliance with the Guide for the Care and Use of Laboratory Animals and were approved by the Institutional Animal Care Committee of the Beijing Institute of Biotechnology (IACUC‐DWZX‐2022‐690). *SIX1‐*floxed KO mice were generated using the Turbo KO approach (Cyagen Biosciences, Inc., Sunnyvale, CA). *SIX1^fl/+^* male and female mice were crossbred to produce SIX1 homozygous mice (*SIX1^fl/fl^*). *SIX1^fl/fl^* mice were bred with Alb‐Cre mice that express hepatocyte‐specific albumin‐Cre recombinase (Alb‐Cre) to generate *SIX1^fl/+^*; Alb‐Cre mice. Finally, the *SIX1^fl/+^*; Alb‐Cre mice were crossed to obtain liver‐specific *SIX1* KO (*SIX1^fl/fl^*; Alb‐Cre) mice. MEFs were isolated from fetal mice tissues and incubated in trypsin solution to obtain a single‐cell suspension. The suspension was then washed twice in DMEM medium and incubated at 37 °C in 5% CO_2_.

### Quantitative Reverse Transcription‐Polymerase Chain Reaction (qRT‐PCR)

Total RNA was extracted using TRIzol reagent according to the manufacturer's instructions (Invitrogen, Carlsbad, CA). Cells were homogenized with TRIzol reagent, vortexed for 1 min with 200 µL chloroform, and centrifuged at 12 000 rpm for 10 min at 4 °C. The upper aqueous phase (containing RNA) was precipitated with isopropanol in equal volume at −80 °C for 1 h and centrifuged at 12 000 rpm for 15 min. RNA pellets were washed with 75% (v/v) ethanol and 100% (v/v) ethanol in succession, air‐dried, and dissolved in 50 µL of nuclease‐free water. Then, 2 µg of total RNA was reverse transcribed into first‐strand cDNA with oligo (dT) primers using Moloney murine leukemia virus reverse transcriptase (Promega, Madison, WI). miRcute Plus miRNA First‐Strand cDNA Kit (Tiangen) was used to transcribe miRNA into cDNA. qPCR was performed in triplicate in 20 µL of reaction mixture containing 10 µL of SYBR Premix Ex Taq Master Mix (2×) (Vazyme, China), 0.5 µM of each primer, and 10 ng cDNA. The relative expression was calculated using the 2^−ΔΔCt^ method. The primers used for real‐time PCR analysis are listed in Table S2 (Supporting Information).

### Western Blot/Immunoblot Analysis

Radioimmunoprecipitation assay buffer (Biomed, China) was used to extract total protein from cells or tissues, and protein quantity was determined by bicinchoninic acid assay. Proteins were transferred to nitrocellulose membranes after SDS‐polyacrylamide gel electrophoresis. The membranes were blocked with 5% skimmed milk for 1 h at room temperature, followed by incubation with primary antibodies including anti‐ACLY (Proteintech; Cat# 67166‐1‐Ig), anti‐ACC1 antibody (Proteintech; Cat# 21923‐1‐AP), anti‐FASN antibody (Proteintech; Cat# 10624‐2‐AP), anti‐SCD1 antibody (Proteintech; Cat# 28678‐1‐AP), anti‐SIX1 antibody (Proteintech; Cat# 10709‐1‐AP), anti‐AIB1 antibody (Santa Cruz Biotechnology; Cat# sc‐9119), anti‐HBO1 antibody (Proteintech; Cat# 13751‐1‐AP), and anti‐β‐actin (Santa Cruz Biotechnology; Cat# sc‐47778HRP) at room temperature for 2 h or overnight at 4 °C. The membranes were washed 3 times with TBST, and incubated with a secondary antibody at room temperature for 2 h, followed by signal detection using an enhanced chemiluminescence kit (Vazyme, China).

### Luciferase Reporter Assay

Luciferase reporter assays were performed according to the manufacturer's instructions (Vigorous, Beijing). Briefly, cells were transfected with 1 µg of promoter luciferase reporter, 0.5 µg of SIX1 expression plasmid or empty vector, and 0.1 µg of a PRL‐TK reporter. Forty‐eight hours after transfection, the cells were harvested, lysed, and centrifuged to obtain supernatants. Twenty microliters of the supernatant were mixed with 100 µL of the Fassay Reagent I and Firefly luciferase. Then add 100 µL of Rassay Reagent II to the bottom of the tube, gently tap the tube wall 3–5 times, mix it well, and put it into the instrument for immediate determination to record the luminescence unit (RLU) of Ranilla luciferase.

### Chromatin Immunoprecipitation (ChIP) and Re‐ChIP

ChIP experiments were performed using the Magna ChIP G Assay Kit (Millipore, Boston, MA) according to the manufacturer's protocol. Briefly, cells were cross‐linked, pelleted, and resuspended in lysis buffer; frozen tissues were cut into 1–3 mm^3^ pieces, cross‐linked and pelleted. The tissues in frozen PBS were broken down by homogenizer, and the precipitation was collected to lysis by cell lysis buffer. The supernatant was collected by cell ultrasound and centrifugation. After incubating supernatants with indicated antibodies and Protein G magnetic beads, the magnetic beads were washed, and the precipitated chromatin complexes were harvested, purified, and de‐crosslinked at 62 °C for 2 h, followed by incubation at 95 °C for 10 min. The precipitated DNA fragments were examined using real‐time PCR. For Re‐ChIP, complexes were eluted from the primary immunoprecipitation by incubation with 10 mM DTT at 37 °C for 30 min, and diluted 1:50 in a buffer [1% Triton X‐100, 2 mM EDTA, 150 mM NaCl, 20 mM Tris/HCl (pH 8.1)], followed by reimmunoprecipitation with the second antibodies and analyzed by real‐time PCR. The PCR primers for re‐ChIP were the same as those for ChIP assays. The primers used for ChIP are listed in Table S3 (Supporting Information).

### Long‐Chain Fatty Acid (LCFA) Assay

Cells (1 × 10^7^) of different treatments were collected into 1.5 mL Eppendorf tubes and then sent to Beijing QLBio Company for LCFA level analysis. A Waters ACQUITY UPLC I‐CLASS chromatography was used for separation. The MS data under negative ion modes were collected by a Waters XEVO TQ‐S Microsystem. The peak area was integrated by the TargetLynx software.

### Measurement of Triglyceride and Total Cholesterol Levels

The levels of triglyceride and total cholesterol were determined by an enzymatic assay kit (Applygen Technologies Inc, Beijing). Briefly, for triglyceride detection, 1 × 10^6^ cells or 50 mg tissues were lysed with 0.1 or 1 mL lysis buffer, respectively, and stood still at room temperature for 10 min. The supernatant of cells or tissues was heated at 70 °C for 10 min, and centrifuged at 2000 rpm at room temperature for 5 min. The supernatant was collected for triglyceride detection using an enzymatic kit (E1013, Applygen Technologies Inc.) following the manufacturer's instructions. For total cholesterol detection, 1 × 10^6^ cells or 100 mg tissues were lysed with 0.1 or 1 mL lysis buffer, respectively, and stood still at room temperature for 10 min. The supernatant of cells or tissues was heated at 70 °C for 10 min, and centrifuged at 2000 rpm at room temperature for 5 min. The supernatant was harvested for total cholesterol detection using an enzymatic kit (E1026, Applygen Technologies Inc.) following the manufacturer's instructions.

### Lipid Droplet Staining

For lipid droplet staining of cells, cells (2 × 10^5^ cells/well) were seeded and cultured on slides in 6‐well plates. Cells were fixed with 4% paraformaldehyde for 15 min, washed twice with PBS, and then stained in the dark for 15 min at 37 °C with BODIPY 493/503 (1 µg mL^−1^). The nuclei were stained using DAPI (5 µg mL^−1^) for 5 min before sealing. Images were obtained using a fluorescence microscope. For lipid droplet staining of liver tissues, tissues were fixed with 4% paraformaldehyde for 24 h at 4 °C. Then the liver tissues were sent to Wuhan Service Company for OCT embedding, frozen section (8 µm), and oil red O staining.

### H&E Staining

Paraffin sections were first dewaxed and watered. The sections were stained with hematoxylin staining solution for 3–5 min, washed with tap water, differentiated by differentiation solution, washed with tap water, returned blue by bluing solution, and washed with running water. The sections were dehydrated with 95% alcohol for 1 min, stained with eosin for 15 sec, and then dehydrated and sealed. Hematoxylin and eosin staining images were collected under a microscope.

### Glucose Tolerance Test (GTT) and Insulin Tolerance Test (ITT)

Before the GTT, mice were fasted overnight (16 h) but allowed free access to water. Baseline (t  = 0) blood samples were taken to measure fasting blood glucose (FBG) before intraperitoneal injection of D (+) – glucose (2 g/kg), and the blood glucose level was recorded after 15, 30, 60, 90 and 120 min with an Accu–Check monitor (Roche Diagnostics, West Sussex, UK) using a drop of blood from the tail vein. After one week, the ITT was performed in overnight‐fasted animals by an intraperitoneal injection of insulin (0.75 U/kg), and the blood glucose level was checked after 15, 30, 60, 90, and 120 min.

### RNA Immunoprecipitation (RIP)

RIP experiments were performed using the Magna RIP RNA‐Binding Protein Immunoprecipitation Kit (Millipore) according to the manufacturer's protocol. Briefly, Wash the cells on the plates twice with 10 mL of ice‐cold PBS. Add 10 mL of ice‐cold PBS. Scrape cells off from each plate and transfer to a centrifuge tube. Collect cells by centrifugation at 1500 rpm for 5 min at 4 °C and discard the supernatant. Re‐suspend the cell pellet in an equal pellet volume of complete RIP Lysis Buffer. Mix by pipetting up and down until the cells have been dispersed and the mixture appears homogeneous. Incubate the lysate on ice for 5 min. Prepare magnetic beads for immunoprecipitation according to the manufacturer's protocol. Add ∼5 µg of the antibody of interest to the magnetic beads tube. Incubate with rotation for 30 min at room temperature. Thaw the RIP lysate (From section I) quickly and centrifuge at 14 000 rpm for 10 min at 4 °C. Remove 100 µL of the supernatant and add to each beads‐antibody complex in RIP Immunoprecipitation Buffer. Remove 10 µL of the supernatant of RIP lysate place it into a new tube and label “input”. Store this input sample at −80 °C until starting RNA purification. Incubate all the tubes by rotating for 3 h overnight at 4 °C. Centrifuge the immunoprecipitation tubes briefly place them on the magnetic separator and discard the supernatant. The beads were washed, and RNA was harvested and reversely transcribed to cDNA for real‐time PCR.

### Cell Proliferation and Invasion

For cell proliferation tests, cells were inoculated in 96‐well plates at a density of 3000 cells per well. Cell proliferation was measured using CCK‐8, 5 times every 24 h, according to the manufacturer's instructions (Dojindo Laboratories, Kumamoto, Japan). Simply, a solution of CCK‐8 was added to each well of cultured cells and placed at 37 °C for 1 h. OD values were measured at 450 nm using a microplate reader. The cell invasion assay was performed using the Matrigel invasion chamber and according to the manufacturer's protocol (BD Biosciences, Franklin Lakes, NJ). Cells (1 × 10^5^) were put on the upper surface of transwell compartments, and DMEM containing 20% fetal bovine serum was added into the lower chamber. After 24 h, transwell compartments were washed with PBS, fixed with 4% polyformaldehyde, and dyed with 0.5% crystal violet. The number of infiltrating cells was measured and photographed in 5 randomly selected microscope fields.

### Analysis of Tumor Growth and Metastasis In Vivo

Animal experiments were approved by the Institutional Animal Care Committee of the Beijing Institute of Biotechnology. Six‐week‐old male nude mice were purchased from Vital River (Beijing, China). To assess tumor growth, 10^7^ differentially treated MHCC97H or HepG2 cells were injected subcutaneously into the BALB/c nude mice. The length and width of the tumor were measured with a caliper at a specified time. The tumor volume was calculated as follows: volume = (longest diameter × shortest diameter^2^)/2. The mice were euthanized at a specified time point. The removed tumor was frozen in liquid nitrogen for further analysis. For lung metastasis analysis, 1 × 10^6^ MHCC97H cells harboring different constructs were injected into the lateral caudal vein of each BALB/c male nude mouse. After 30 days, the lungs were removed for metastatic measurements.

### Human Clinical Samples, Fluorescence In Situ Hybridization (FISH), and Immunohistochemistry (IHC)

Samples of 73 patients with liver cancer and matched normal liver tissues were obtained from the Chinese PLA General Hospital, with the informed consent of patients and approval of the Institutional Review Committees of the Chinese PLA General Hospital (S2016‐098‐01). The expression level of miR‐145‐5p and DGUOK‐AS1 was determined following miRNA FISH and lncRNA FISH instructions (Exonbio), respectively. The FISH probes were synthesized and labelled with digoxin by Exonbio, and the sequences were as follows: AGGGATTCCTGGGAAAACTGGAC (miR‐145‐5p); TGTTCTCTGAGTAAGACTTGGCGAGTATGTGA (DGUOK‐AS1‐1); GTTCAGCAGCATCCAACAGTGTTAGTTCCA (DGUOK‐AS1‐2); TTGAGGCTCTGATGAACATTCCAGTGCTG (DGUOK‐AS1‐3); CCGTGCGGAATCAACTTCAGCTCATCAAT (DGUOK‐AS1‐4); TGAAGAACCACAGGCTTGCACATCCAATTATG (DGUOK‐AS1‐5); AAAGCCTCCTTATTTCGCACCCTCCATG (DGUOK‐AS1‐6); GAGTTCTTAGGGCTTCCTCCTCTAGCAAGG (DGUOK‐AS1‐7). The scramble probe (negative control) sequence was GTGTAACACGTCTATACGCCCA. The levels of SIX1 and SCD1 expression were determined by IHC and cyanine 3 system (K1051, APExBIO). IHC of specimens was analyzed as previously described.^[^ ^50^ ^]^ The fluorescence intensity was examined using a microscope (BX53F; Olympus, Tokyo, Japan). The DGUOK‐AS1, miR‐145‐5p, SIX1 and SCD1 score was calculated by multiplying staining intensity (1, low; 2, medium; 3, strong) by stained cells percentage (0–100%).

### Statistical Analysis

Trial experiments or similar experiments performed previously were used to estimate the sample size with adequate statistical power. Data were presented as mean ± standard deviation (SD) (*n* = 3, unless otherwise specified). The Student's *t‐*test was used to compare the means of 2 groups. When more than 2 groups were compared, a one‐way ANOVA was performed. Comparisons were performed using independent sample *t‐tests* and Mann‐Whitney U tests for parametric and non‐parametric data, respectively. The correlation of expression among DGUOK‐AS1, miR‐145‐5p, SIX1, and SCD1 was determined using Spearman's Rank Correlation test. Disease‐free survival (DFS) and overall survival (OS) were estimated using the Kaplan‐Meier method, and differences between survival curves were examined using the log‐rank test. All statistical tests were two‐sided. Statistical analyses were performed using SPSS Statistics 21 and GraphPad Prism 9. A *p*‐value of <0.05 was considered statistically significant.

## Conflict of Interest

The authors declare no conflict of interest.

## Author Contributions

L.L., X.Z., and G.X. contributed equally to this work. Q.Y. and Y.Z. conceived the project, designed the experiments, and analyzed the data. L.L., X.Z., and G.X. designed and performed the experiments and analyzed the data, aided by R.X., S.L., S.W., Y.L., Y.Y., and S.G. J.L. and G.L. collected clinical samples and analyzed the data. Q.Y., L.L., and X.Z. wrote the manuscript.

## Supporting information

Supporting Information

## Data Availability

The data that support the findings of this study are available from the corresponding author upon reasonable request.
