# Correction to “Transcriptional Regulation of *De Novo* Lipogenesis by SIX1 in Liver Cancer Cells”

**DOI:** 10.1002/advs.75221

**Published:** 2026-04-10

Li L, Zhang X, Xu G, Xue R, Li S, Wu S, Yang Y, Lin Y, Lin J, Liu G, Gao S, Zhang Y, Ye Q. “Transcriptional Regulation of *De Novo* Lipogenesis by SIX1 in Liver Cancer Cells”. Adv Sci (Weinh). 2024, 11(41):e2404229. doi: 10.1002/advs.202404229.

In Figure 6F of the original article, there was an error in the lung H & E staining for the antagomiR‐145‐5p sample, which was an inadvertent duplication of the DGUOK‐AS1 sample. The corrected figure, based on the original source data, is provided below.


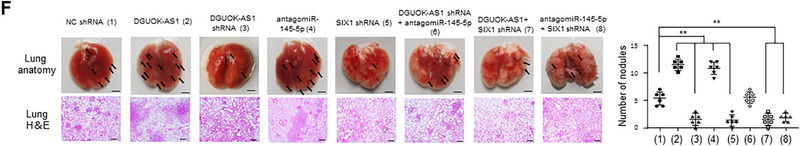


We apologize for this error.

