# Supplementary material for: Transcriptional Regulation of De Novo Lipogenesis by SIX1 in Liver Cancer Cells
Source: Adv Sci (Weinh). 2024 Sep 11;11(41):2404229. doi: 10.1002/advs.202404229 (PMC11538671; doi:10.1002/advs.202404229)
Supplement: Supplementary file 1 — Supporting Information [file ADVS-11-2404229-s001.docx]

Supporting Information

**Transcriptional Regulation of *De Novo* Lipogenesis by SIX1 in Liver Cancer Cells**

*Ling Li, Xiujuan Zhang, Guang Xu, Rui Xue, Shuo Li, Shumeng Wu, Yuanjun Yang, Yanni Lin, Jing Lin, Guoxiao Liu, Shan Gao, Youzhi Zhang^*^, Qinong Ye^*^.*

**Figure S1**


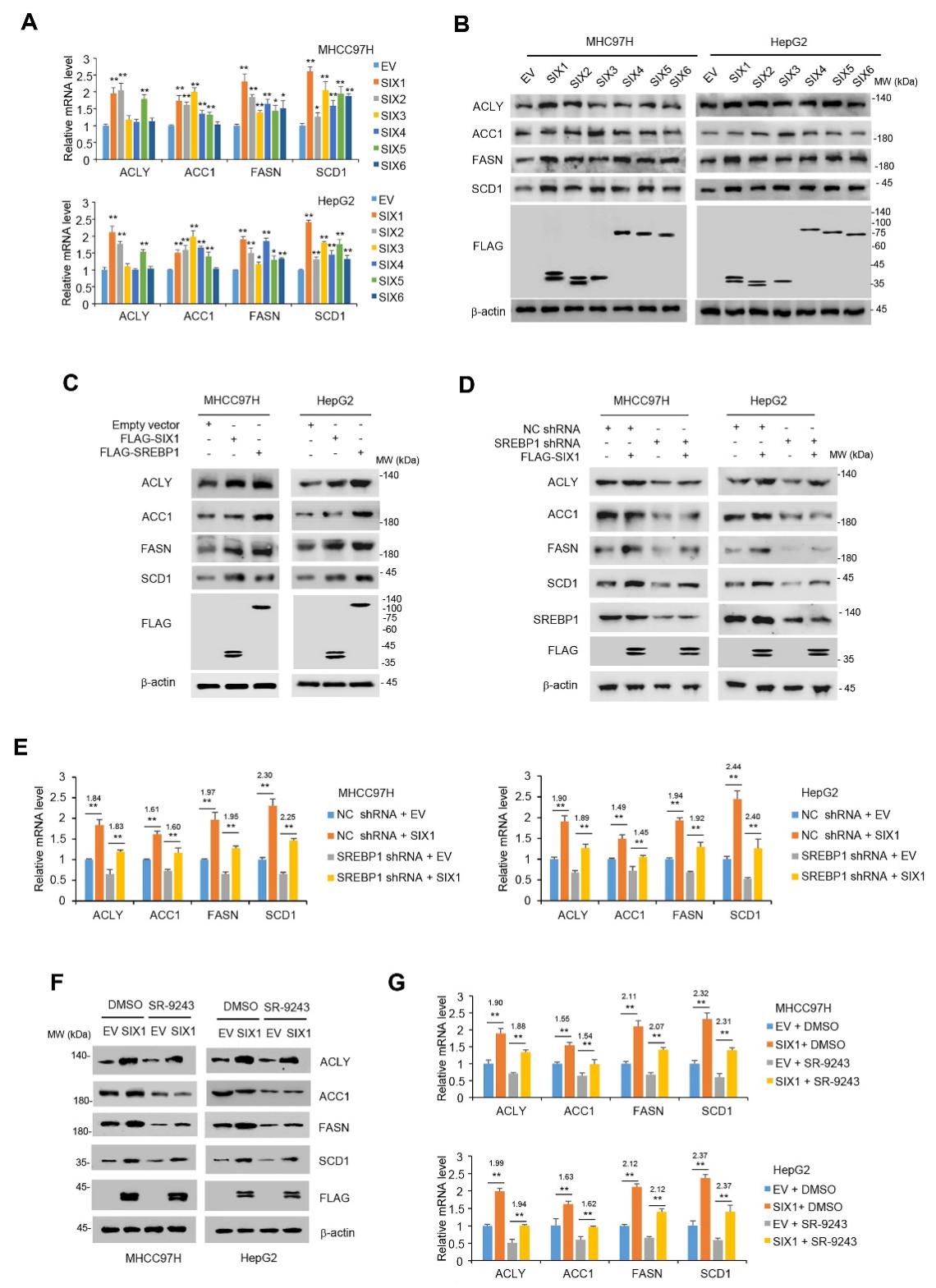


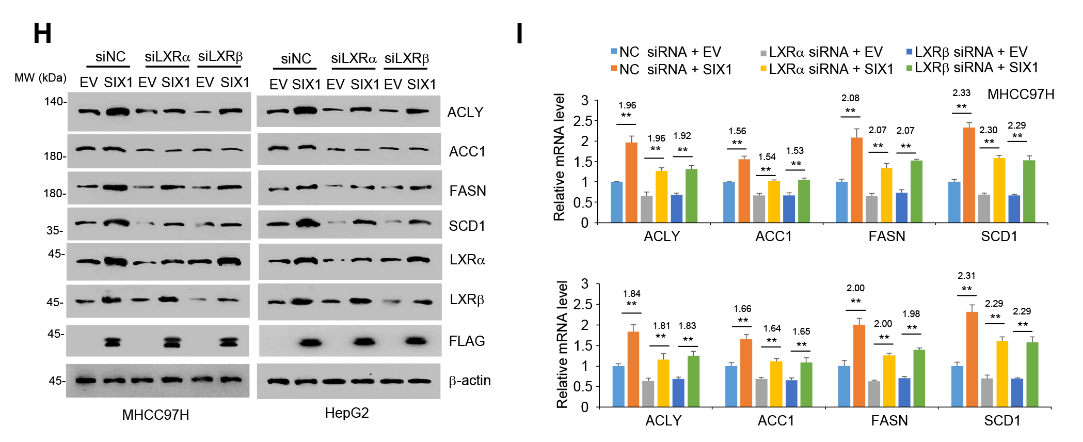


**Figure S1. SIX family members regulate de novo lipogenesis-related gene expression. A, B) qRT-PCR (A)** and immunoblot **(B)** analysis of MHCC97H and HepG2 cells transiently transfected with empty vector (EV), Flag-tagged SIX family members. MW, molecular weight. **C)** Immunoblot analysis of MHCC97H and HepG2 cells transiently transfected with EV, FLAG-tagged SIX1 or FLAG-tagged SREBP1. **D, E)** MHCC97H and HepG2 cells stably expressing SREBP1 shRNA or negative control (NC) shRNA were transiently transfected with Flag-tagged SIX1 or EV. Lipogenic gene expression was examined using immunoblot (D) and qRT-PCR (E). **F, G)** Immunoblot (F) and qRT-PCR (G) analysis of MHCC97H and HepG2 cells transfected with FLAG-tagged SIX1 or EV and treated with or without the LXRs inhibitor SR-9243 (1μM). **H, I)** MHCC97H and HepG2 cells stably infected with lentivirus carrying FALG-SIX1 or EV were transiently transfected with LXRα siRNA (siLXRα), LXRβ siRNA (siLXRβ) or NC siRNA (siNC). The expression of lipogenesis-related genes was examined using immunoblot (H) and qRT-PCR (I). Data shown are mean ± SD of triplicate measurements. Experiments have been repeated three times with similar results. Two-sided Student’s t-test was used to compare the means of two groups. When more than two groups were compared, one-way ANOVA was performed. ^*^*p* < 0.05, ^**^*p* < 0.01 versus corresponding control. Average fold change versus corresponding control is indicated (E, G, I).

**Figure S2**


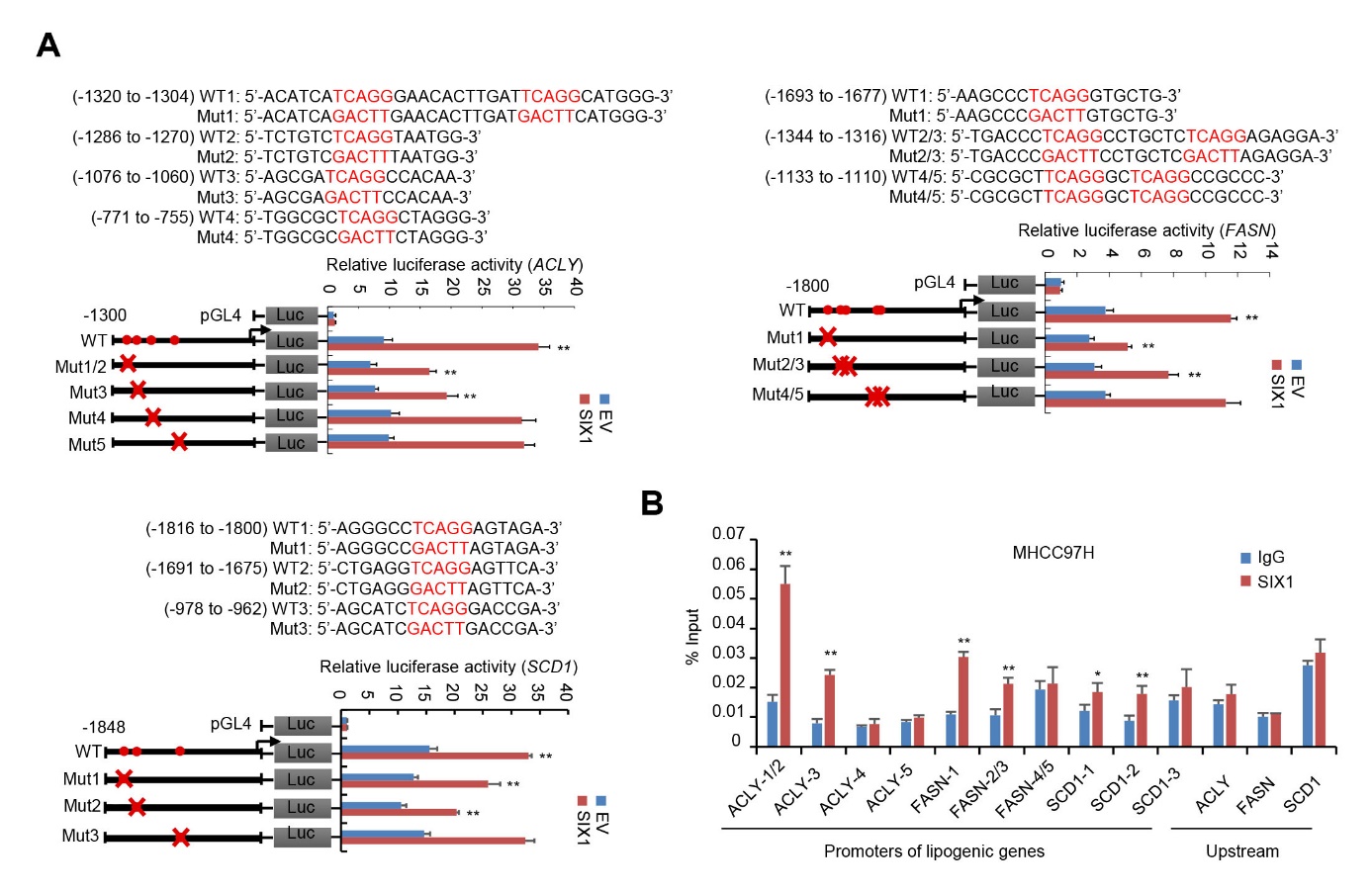


**Figure S2. SIX1 binds SIX1-responsive element to increase lipogenesis-related gene promoter activity.** **A)** Luciferase activity of different lipogenesis-related gene promoter reporters in MHCC97H cells transfected with FLAG-tagged SIX1 or EV. Filled circles show the position of the putative SIX1-binding sites, and the “X” shows the mutated SIX1-binding sites. The red letters of each binding region indicate the putative or mutated SIX1-binding sequences. WT, wild-type. Mut, mutant. **B)** ChIP analysis of SIX1 occupancy on promoters of lipogenesis-related genes in MHCC97H cells. IgG, normal serum. The different number after each gene represents the regions containing different putative SIX1-binding sites from left to right shown in (A). Data shown are mean ± SD of triplicate measurements. Experiments have been repeated three times with similar results. Data were analyzed using two-tailed Student’s t test. ^*^*p* < 0.05, ^**^*p* < 0.01 versus respective promoter reporter with EV (A). ^**^*p* < 0.01 versus respective normal IgG (B).

**Figure S3**


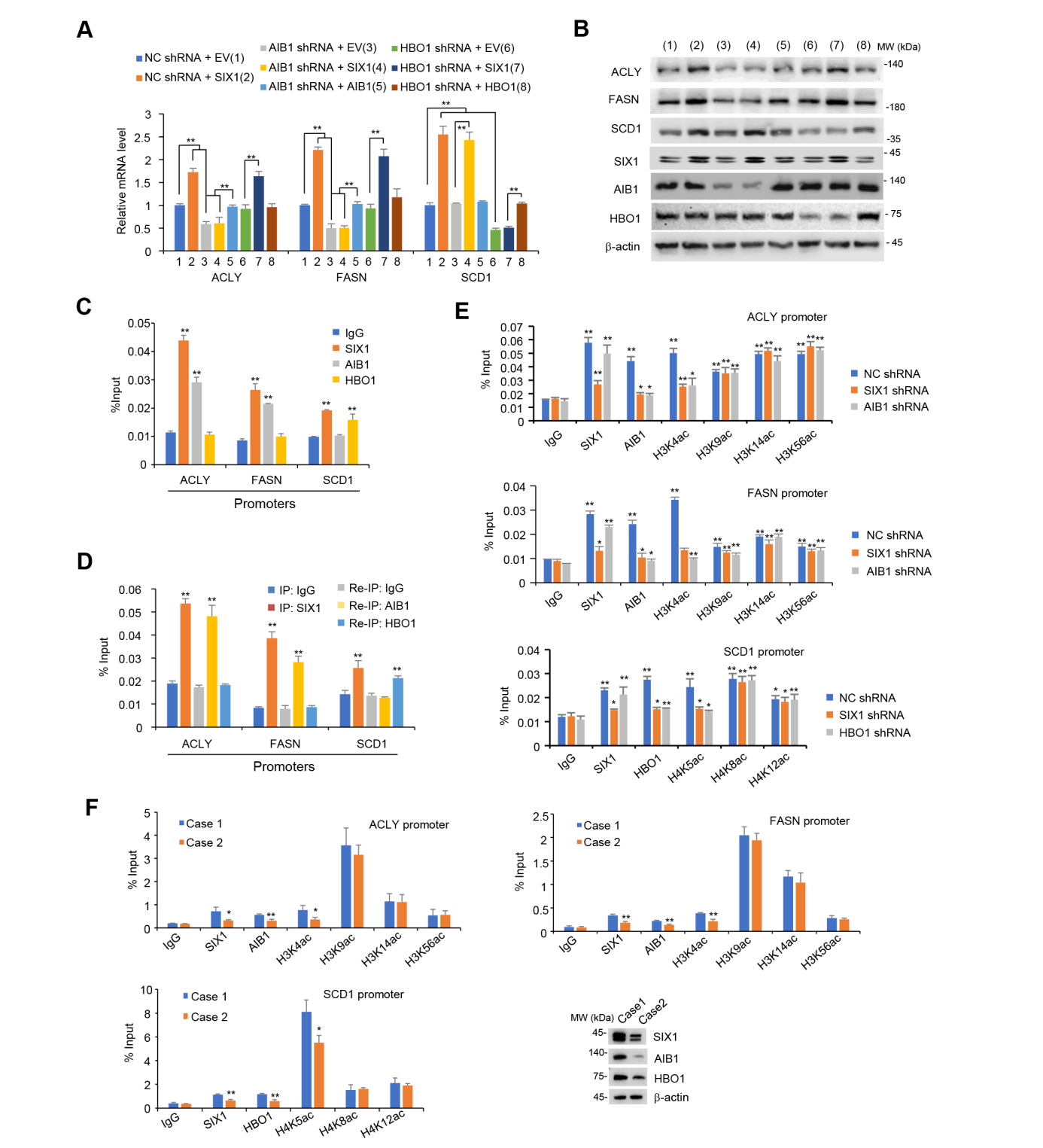


**Figure S3. SIX1 promotes lipogenic gene expression via AIB1 and HBO1. A, B)** MHCC97H cells stably infected with lentivirus carrying NC shRNA, AIB1 shRNA or HBO1 shRNA were transfected with SIX1, HBO1, AIB1 or EV as indicated. Lipogenic gene expression was examined using qRT-PCR (A) and immunoblot (B). **C)** ChIP analysis of SIX1, HBO1 and AIB1 occupancy on the promoters of lipogenic genes in MHCC97H cells. Promoter regions of each gene represent the region containing the first or second SIX1 binding site shown in Figure 2B within the gene promoters analyzed. **D)** Re-ChIP analysis of the occupancy of SIX1 and HBO1/AIB1 on the indicated lipogenic gene promoters in MHCC97H cells. **E)** ChIP analysis of SIX1, HBO1, AIB1 and H3 and H4 acetylation (ac) occupancy on the indicated promoters of lipogenic genes in MHCC97H cells stably infected with lentivirus carrying SIX1 shRNA, HBO1 shRNA, AIB1 shRNA or NC shRNA. **F)** ChIP analysis of SIX1, AIB1, HBO1 and H3 and H4 acetylation occupancy on the indicated promoters of lipogenic genes in two cases of liver cancer tissues. The expression level of SIX1, HBO1 and AIB1 in two cases of liver cancer tissues were detected by immunoblot. Data shown are mean ± SD of triplicate measurements. Experiments have been repeated three times with similar results. Two-sided Student’s t-test was used to compare the means of two groups. When more than two groups were compared, one-way ANOVA was performed. ^*^*p* < 0.05, ^**^*p* < 0.01 versus respective WT MHCC97H cells transfected with NC shRNA (A). ^*^*p* < 0.05, ^**^*p* < 0.01 versus respective normal IgG (C-E). ^*^*p* < 0.05, ^**^*p* < 0.01 versus Case 1 (F).

**Figure S4**


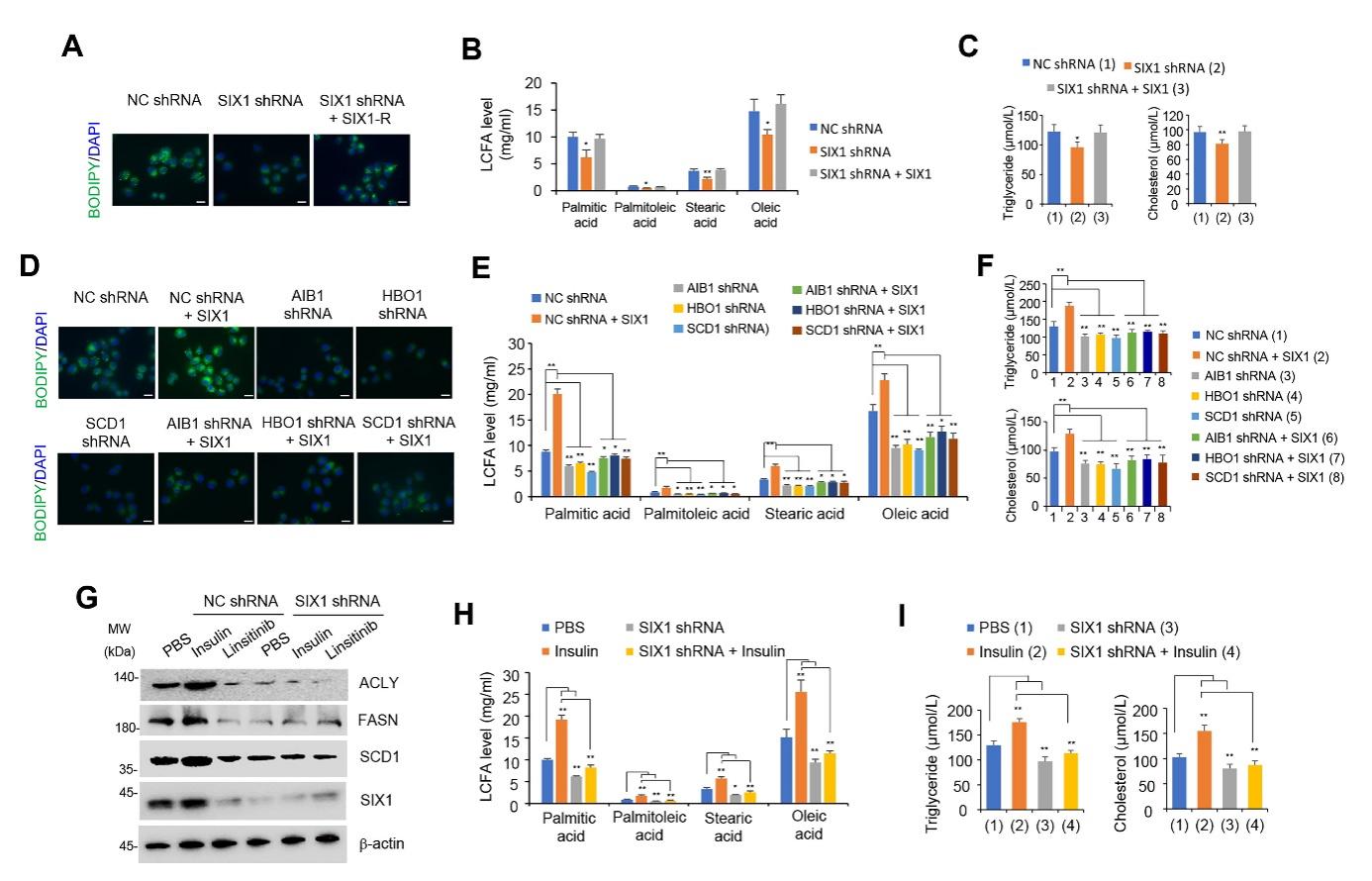


**Figure S4. SIX1 is an insulin responsive gene and promotes lipogenesis. A)** Lipid droplets (LDs) were visualized using BODIPY 493/503 in MHCC97H cells stably expressing SIX1 shRNA or NC shRNA or SIX1 shRNA-expressing MHCC97H cells transfected with SIX1-R. **B)** Analysis of long-chain fatty acid levels by liquid chromatography-mass spectrometry in cells from (A). **C)** Analysis of triglyceride and cholesterol levels in in cells from (A). **D-F)** Analysis of LDs (D), long-chain fatty acid levels (E) and triglyceride and cholesterol levels (f) in NC shRNA- or HBO1/AIB1 shRNA-expressing MHCC97H cells transfected with SIX1 or EV. **G)** Immunoblot analysis of SIX1 shRNA- or NC shRNA-expressing MHCC97H cells treated with insulin (100 nM) or linsitinib (1 µM). **H, I)** Analysis of long-chain fatty acids levels (H) and triglyceride and cholesterol levels (I) in cells from (G). Data shown are mean ± SD of triplicate measurements (B, E, H). Data shown are mean ± SD of quintuplicate measurements (C, F, I). Experiments have been repeated three times with similar results. Two-sided Student’s t-test was used to compare the means of two groups. When more than two groups were compared, one-way ANOVA was performed. ^*^*p* < 0.05, ^**^*p* < 0.01 versus NC shRNA (B, C). ^*^*p* < 0.05, ^**^*p* < 0.01 (E, F, H, I).

**Figure S5**


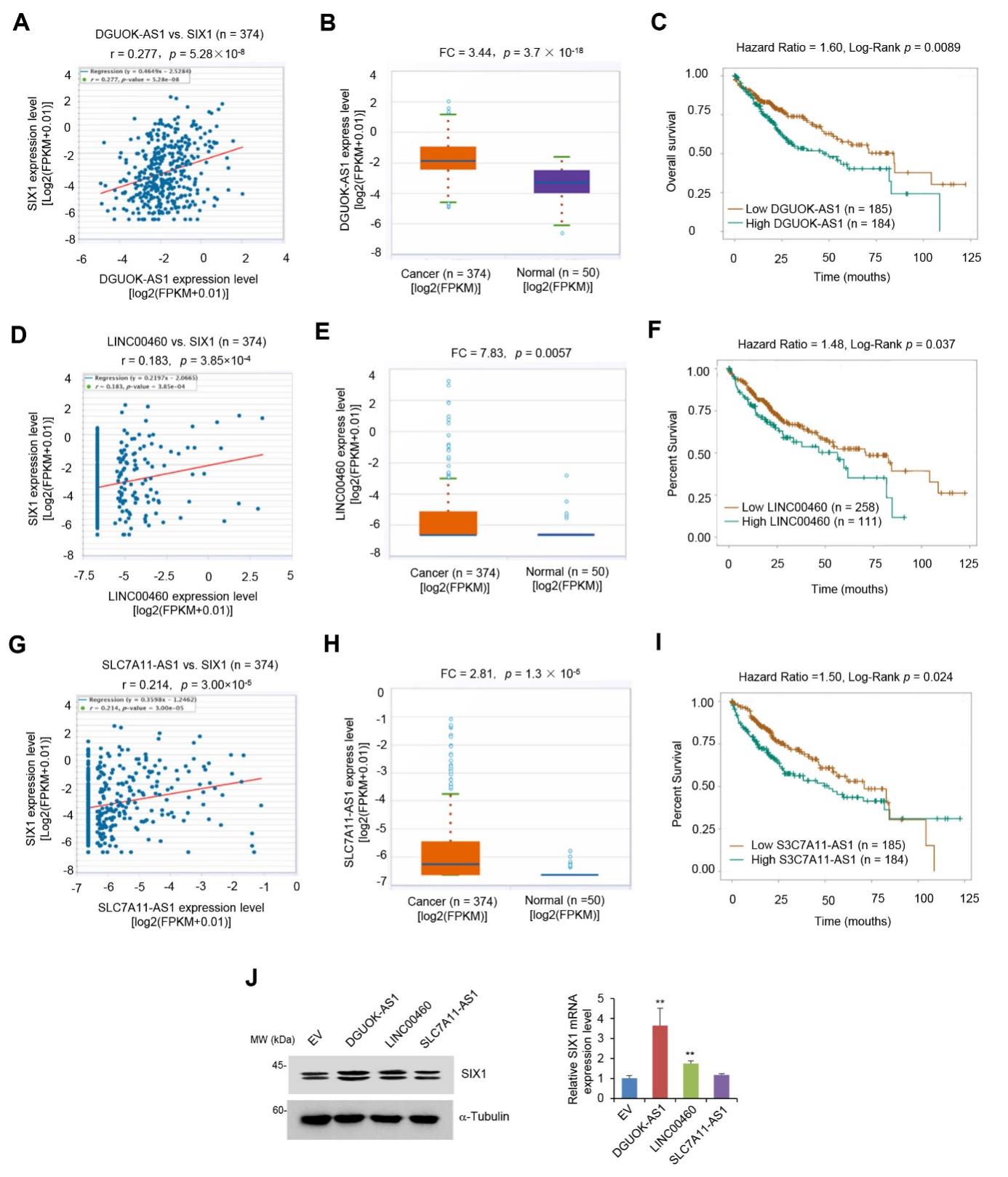


**Figure S5. Prediction of lncRNAs upregulated and positively correlated with SIX1 in liver hepatocellular carcinoma (LIHC). A)** Correlation of DGUOK-AS1 expression with SIX1 expression in LIHC patients was analyzed using ENCORI database (https://starbase.sysu.edu.cn/). **B)** Comparison of DGUOK-AS1 expression between LIHC and normal samples using ENCORI database. FC, fold change. **C)** Overall survival curves for DGUOK-AS1 expression in LIHC patients was plotted based on ENCORI database. **D)** Correlation of LINC00460 expression with SIX1 expression in LIHC patients was analyzed using ENCORI database. **E)** Comparison of LINC00460 expression between LIHC and normal samples using ENCORI database. **F)** Overall survival curves for LINC00460 expression in LIHC patients was plotted based on ENCORI database. **G)** Correlation of SLC7A11-AS1 expression with SIX1 expression in LIHC patients was analyzed using ENCORI database. **H)** Comparison of SLC7A11-AS1 expression between LIHC and normal samples using ENCORI database. **I)** Overall survival curves for SLC7A11-AS1 expression in LIHC patients was plotted based on ENCORI database. **J)** Immunoblot (left) and qRT-PCR (right) analysis of HepG2 cells transfected with the indicated lncRNAs or EV. Statistical significance was assessed by one-way ANOVA. Experiments have been repeated three times with similar results. Data were analyzed using two-tailed Student’s t test. ^**^*p* < 0.01 versus EV.

**Figure S6**


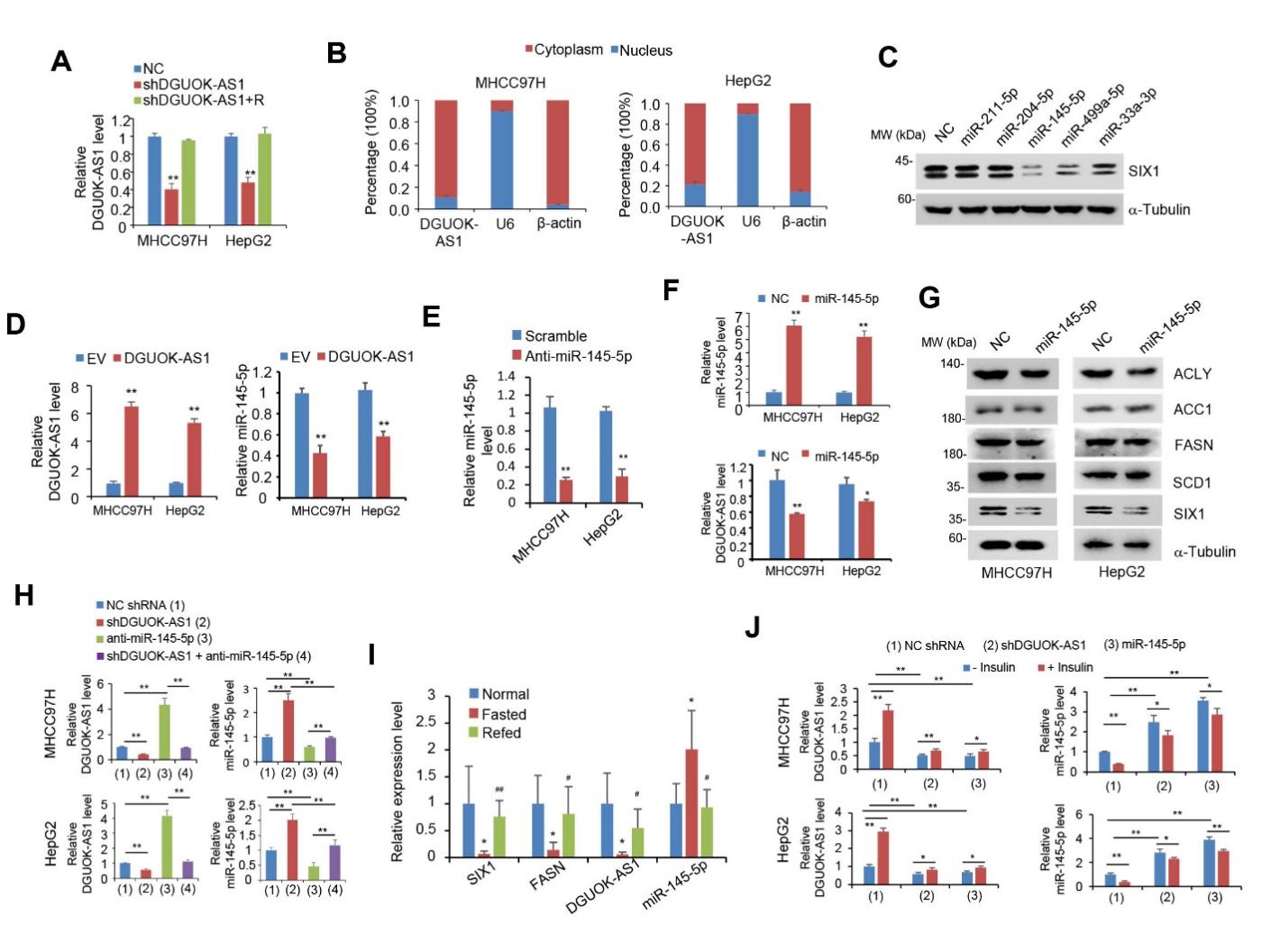


**Figure S6. DGUOK-AS1/miR-145-5p axis regulates expression of SIX1 and de novo lipogenic genes. (A)** qRT-PCR analysis of DGUOK-AS1 expression in cells from Figure 5A. **(B)** qRT-PCR analysis of DGUOK-AS1 expression in the cytoplasm and nucleus of MHCC97H and HepG2 cells. U6 and β-actin were used as the nuclear and cytoplasmic marker, respectively. **(C)** Immunoblot analysis of SIX1 expression in HepG2 cells transfected with the indicated miRNAs. **(D)** qRT-PCR analysis of miR-145-5p expression in MHCC97H and HepG2 cells transfected with EV or DGUOK-AS1. **(E)** qRT-PCR analysis of miR-145-5p expression in cells from Figure 5C. **(F)** qRT-PCR analysis of DGUOK-AS1 and miR-145-5p expression in MHCC97H and HepG2 cells transfected with NC or miR-145-5p mimics. **(G)** Immunoblot analysis of MHCC97H and HepG2 cells transfected with miR-145-5p mimics or NC. **(H)** qRT-PCR analysis of DGUOK-AS1 and miR-145-5p expression in cells from Figure 5D. **(I)** C57BL/6J mice were fed a normal diet, subjected to fasting or fasted and then refed (n = 4 per group). Livers were collected and analyzed by qRT-PCR. **(J)** qRT-PCR analysis of DGUOK-AS1 and miR-145-5p expression in cells from Figure 5r. Data shown are mean ± SD of triplicate measurements. Experiments have been repeated three times with similar results. Two-sided Student’s t-test was used to compare the means of two groups. When more than two groups were compared, one-way ANOVA was performed. ^*^*p* < 0.05, ^**^*p* < 0.01 versus corresponding control (A, D, E, F). ^*^*p* < 0.05 versus normal diet; ^#^*p* < 0.05, ^##^*p* < 0.01 versus fasting (I). ^*^*p* < 0.05, ^**^*p* < 0.01 (H, J).

**Figure S7**


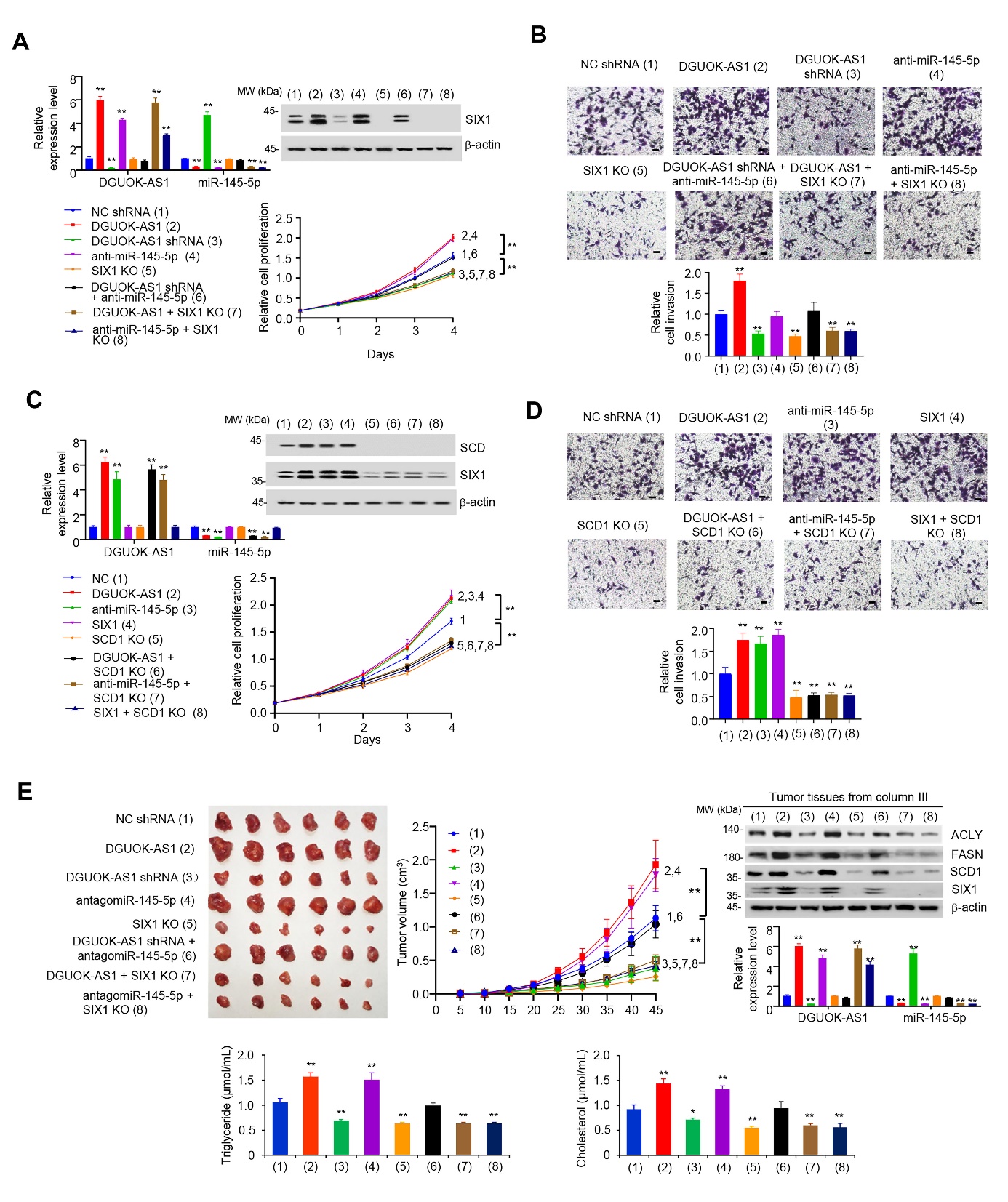


**Figure S7. The DGUOK-AS1/miR-145-5p/SIX1 axis modulates HCC growth and metastasis. A)** HepG2 cells stably expressing DGUOK-AS1 shRNA, SIX1 KO or NC shRNA were transiently transfected with DGUOK-AS1 or anti-miR-145-5p as indicated, and the cell proliferation curve was then determined. **B)** Cell invasion assay of HepG2 cells as in (A). The relative cell invasions are shown in the lower panel. Scale bar, 100 μm. **C)** HepG2 cells stably expressing SCD1 shRNA or NC shRNA were transiently transfected with DGUOK-AS1, anti-miR-145-5p or SIX1 as indicated, and the cell proliferation curve was then determined. **D)** Cell invasion assay of HepG2 cells as in (C). The relative cell invasions are shown in the lower panel. Scale bar, 100 μm. E) Tumor growth curve of HepG2 cells stably expressing DGUOK-AS1, DGUOK-AS1 shRNA, SIX1 shRNA or NC shRNA and treated with antagomiR-145-5p as indicated (n = 6). Images of xenograft tumors are shown in the left panel. Representative immunoblot shows expression of de novo lipogenesis-related gene in representative tumor tissues. Triglyceride and cholesterol levels were measured in representative tumor tissues. Data shown are mean ± SD of triplicate measurements. Experiments have been repeated three times with similar results. Statistical significance was assessed by one-way ANOVA. ^*^*p* < 0.05, ^**^*p* < 0.01 versus corresponding control.

**Figure S8**


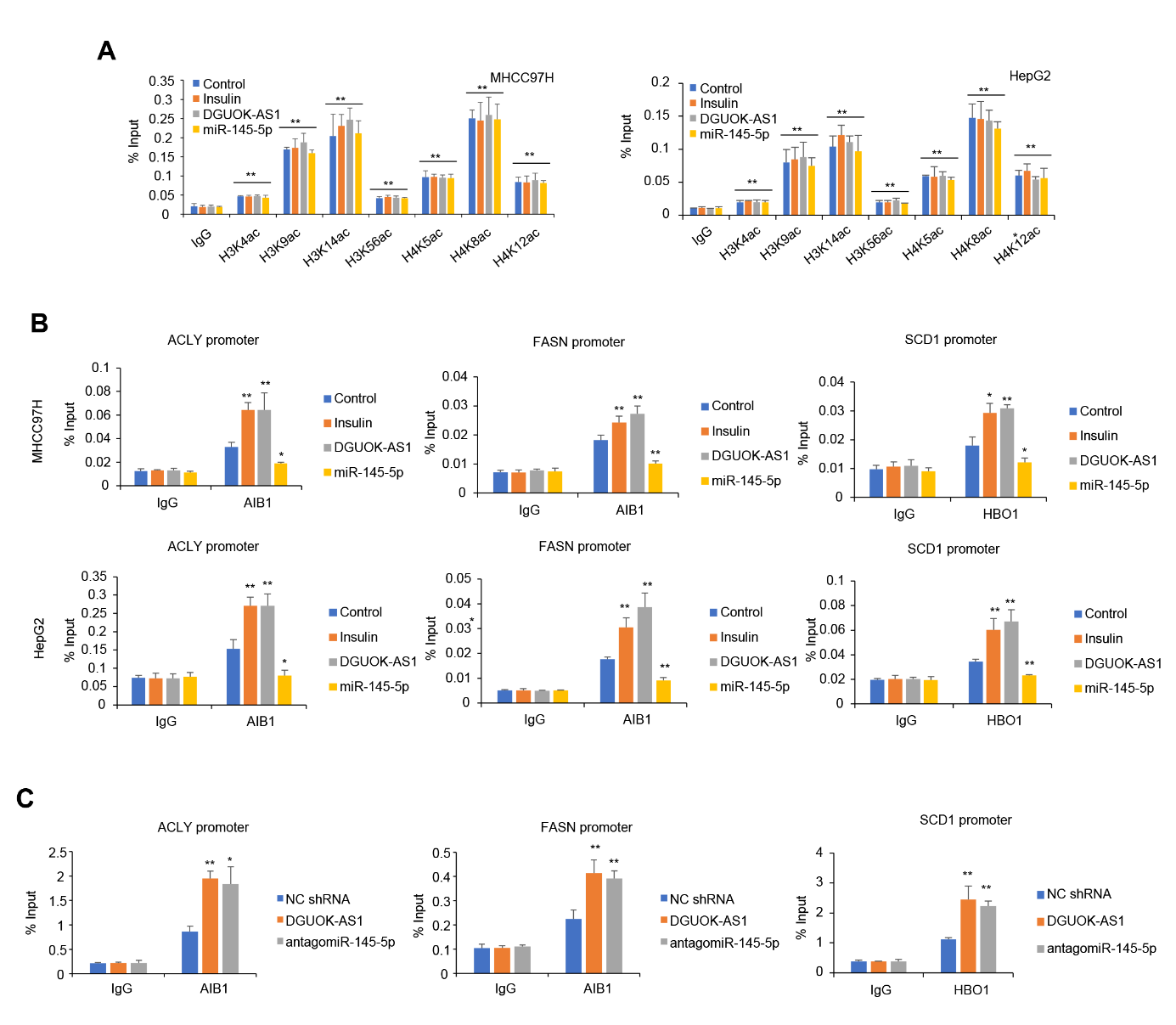


**Figure S8. Insulin/DGUOK-AS1/miR-145-5p axis alters the binding of AIB1 and HBO1 to the DNL-related gene promoters. A)** ChIP analysis of H3 and H4 acetylation occupancy on the SIX1 promoter in MHCC97H or HepG2 cells treated with insulin (100 nM) or transfected with DGUOK-AS1 or miR-145-5p. **B)** ChIP analysis of AIB1 and HBO1 occupancy on the indicated promoters of lipogenic genes in MHCC97H or HepG2 cells treated with insulin (100 nM) or transfected with DGUOK-AS1 or miR-145-5p. **C)** ChIP analysis of AIB1 and HBO1 occupancy on the indicated promoters of lipogenic genes in the indicated tumor tissues in Figure S7E. Data shown are mean ± SD of triplicate measurements. Experiments have been repeated three times with similar results. Two-sided Student’s t-test was used to compare the means of two groups. When more than two groups were compared, one-way ANOVA was performed. ^**^p < 0.01 versus respective normal IgG (A). ^*^*p* < 0.05, ^**^*p* < 0.01 versus corresponding control (B). ^*^*p* < 0.05, ^**^*p* < 0.01 versus corresponding NC shRNA (C).

**Table S1. The cDNA target sequences of shRNAs or siRNAs**

| **Gene** | **Target sequence (5’→3’)** |
| --- | --- |
| SIX1 (siRNA)  HBO1 (siRNA)  AIB1 (siRNA) ^[1]^  LXRα ^[2]^  LXRβ ^[2]^  SIX1 (shRNA)  HBO1 (shRNA)  SCD1 (shRNA) ^[3]^  DGUOK-AS1(shRNA)  SREBP1 (shRNA) ^[4]^ | TCGATGCTGCCGTCGTTTGGCTTTA  CGAAGGAAGAGGAATGCAGGCAGTA  GGTGAATCGAGACGGAAAC  GAAGAACAGAUCCGCUUGATT  UGAGGAGCAGAUUCGGAAGTT  AGAACGAGAGCGTACTCAA  ACCGGTCATTCATTAGGCC  ATATCAGGGCGAATGTCGTCT  GGAGATTTCCCTTTCTAGTCC  CCACCGTTTCTTCGTGGAT |

**References:**

[1] Oh A, List HJ, Reiter R, et al. The nuclear receptor coactivator AIB1 mediates insulin-like growth factor I-induced phenotypic changes in human breast cancer cells. Cancer Res 2004;64:8299-8308.

[2] Lee JH, Park SM, Kim OS, et al. Differential SUMOylation of LXRα and LXRβ Mediates Transrepression of STAT1 Inflammatory Signaling in IFN-γ-Stimulated Brain Astrocytes. Mol Cell 2009;35:806-17.

[3] Li H, Chen Z, Zhang Y, et al. MiR-4310 regulates hepatocellular carcinoma growth and metastasis through lipid synthesis. Cancer Lett 2021;519:161-171.

[4] Li C, Peng X, Lv J, et al. SREBP1 as a potential biomarker predicts levothyroxine efficacy of differentiated thyroid cancer. Biomed Pharmacother 2020;123:109791.

**Table S2. Primers used for real-time PCR**

| **Gene** | **Species** | **Forward (5’→3’)** | **Reverse (5’→3’)** |
| --- | --- | --- | --- |
| ACLY  ACC1  FASN  SCD1  DGUOK-AS1  SIX1  α-Tubulin  β-actin  miR-145-5p  U6  ACLY  ACC1  FASN  SCD1  α -Tubulin  β -actin | Human  Human  Human  Human  Human/Mouse  Human  Human  Human  Human/Mouse  Human/Mouse  Mouse  Mouse  Mouse  Mouse  Mouse  Mouse | CAgCAggACAgCATCTTTTTC  CTCTCACgCTCAAgTCACCA  ACTCCATgTTTggTgTTTgTC  CCCCACCTACAAggATAAggA  TGCTCCCAGAACTCTAACCC  CGCGCACAATCCCTACCCATCGCC  CCAAGCTGGAGTTCTCTA  TCGTGCGTGACATTAAGGAG  GTCCAGTTTTCCCAGGAATCCT  CGCGCTTCGGCAGCACATATACT  GCCAGCGGGAGCACATC  TGACAGACTGATCGCAGAGAAAG  GCTGCGGAAACTTCAGGAAAT  TTCTTCTCTCACGTGGGTTG  AACCAGATGGTGAAATGTGACCCT  CCACAGCTGAGAGGGAAATC | TggACTTgggACTgAATCTTg  ACTTggTTATggCgAAgCTC  TggAgATCACATgCggTTTA  CACgAgCCCATTCATAgACAT  CACCCACTCTTGAGCCACTT  CTTCCAGAGGAGAGAGTTGGTTCTG  CAATCAGAGTGCTCCAGG  ATGCCAGGGTACATGGTGGT  General reverse primer (Tiangen)  ACGCTTCACGAATTTGCGTGTC  CTTTGCAGGTGCCACTTCATC  TGGAGAGCCCCACACACA  AGAGACGTGTCACTCCTGGACTT  CGGGCTTGTAGTACCTCCTC  CACAGTGGGAGGCTGGTAGTTAAT  AAGGAAGGCTGGAAAAGAGC |

**Table S3. Primers used for ChIP**

| **Gene** | **Forward (5’→3’)** | **Reverse (5’→3’)** |
| --- | --- | --- |
| ACLY-1/2  ACLY-3  ACLY-4  ACLY-5  FASN-1  FASN-2/3  FASN-4/5  SCD1-1  SCD1-2  SCD1-3  ACLY-Upstream  FASN-Upstream  SCD1-Upstream  SIX1 | GGACAAGAGAAGAGAGGTGATAAG  ATTCTGCCCCACAAGTTCACCAGC  GTTACAGCCAGTTGGGCTAATCCT  TGGATGGGCCAGCGGGACTACAA  CAAAGTGCTGGGATTACAGGCGTG  TTCCCTTGTCCTTCCTTGACCCT  GCAAGCGCGAAGCGGTCAGAAAAG  TTGTTCCTTTTGCTGAGGTAGGGC  CCTGTAATCCCAGCACTTTGGGAG  CAGTATTTGGGAAAGACATGGGCA  TTTTGCCATGTTGGCCAGGCTGGT  AATATAGACAGTGCCATCGGCTGG  GTGACAGCTGAGCAGCCTTGACTT  AGCAGAGGTGGCACTGCAA AACAC | AATCCTGTTGCTGCCAGGAGTAAG  AGACAGTCTCTTGTTCCCACCAAC  CGGGCTAGGCTGGGAGGAGAAC  GATTGGCCACACGCGTTCCCTAG  TGGCTCCCAAGGAGGTTGGAGACT  CACAGCCCCAGAAGGGAAAAGAC  CTCCCGAGTGATTCCTCGAATCGT  CTGGCCCGGAGAATATTTTCATTC  CCTGTAATCCCAGCACTTTGGGAG  CAGAAACACCATATGAGAGCCCAAG  TCACTTGAGCCCAGGAGGTCAAAG  ATGGAGCAAGTGACGGAGGCTAAC  GGCAACAGAGTGATACCCTGTTTC  CCCTCGTTCACAAAAGACCT CCAT |
